# Tormentil Rhizome Ethanolic Extract and Its Gut Microbiota-Derived Metabolites: Modulation of Tight Junction Integrity and Anti-Inflammatory Potential in Caco‑2 Cells

**DOI:** 10.1021/acs.jafc.6c03087

**Published:** 2026-07-19

**Authors:** Aleksandra Kruk, Łukasz Grześkowiak, Inna Vlasova, Yuliia Kostenko, Jürgen Zentek, Sebastian Granica, Jakub P. Piwowarski

**Affiliations:** † Department of Pharmaceutical Biology, Faculty of Pharmacy, 37803 Medical University of Warsaw, Banacha 1b Street, Warsaw 02-097, Poland; ‡ Institute of Animal Nutrition, 9166 Freie Universitat Berlin, Konigin-Luise 49 Street, Berlin 14195, Germany; § Department of Pharmaceutical Microbiology and Bioanalysis, Faculty of Pharmacy, Medical University of Warsaw, Banacha 1b Street, Warsaw 02-097, Poland

**Keywords:** alcoholic beverages, dietary supplements, functional foods, tight junction, human intestinal epithelium, gut microbiota-derived metabolites, tormentil rhizome

## Abstract

Disruption of intestinal barrier integrity contributes to inflammation, infection, and chronic disease. Tormentil rhizome ethanolic extract (TR-EtOH), traditionally used in functional alcoholic beverages, is rich in polyphenols that undergo extensive gut microbiota metabolism. This study evaluated the phytochemical composition, total phenolic content, antioxidant activity, and barrier-protective effects of TR-EtOH and its gut-derived metabolites (TREMs). Microbiota metabolism markedly reduced phenolic content and antioxidant activity, consistent with the degradation of polymeric tannins. Biological activity was investigated in a *Clostridioides difficile* toxin-induced Caco-2 model using TEER, qPCR, Western blot, and cytokine secretion assays. TR-EtOH preserved epithelial integrity and reduced inflammatory responses, whereas TREMs showed donor-dependent effects on barrier stabilization and cytokine modulation. These findings indicate that native tormentil polyphenols and microbiota-derived metabolites may protect the intestinal barrier through complementary mechanisms, supporting their potential use in functional food and beverage development.

## Graphical abstract

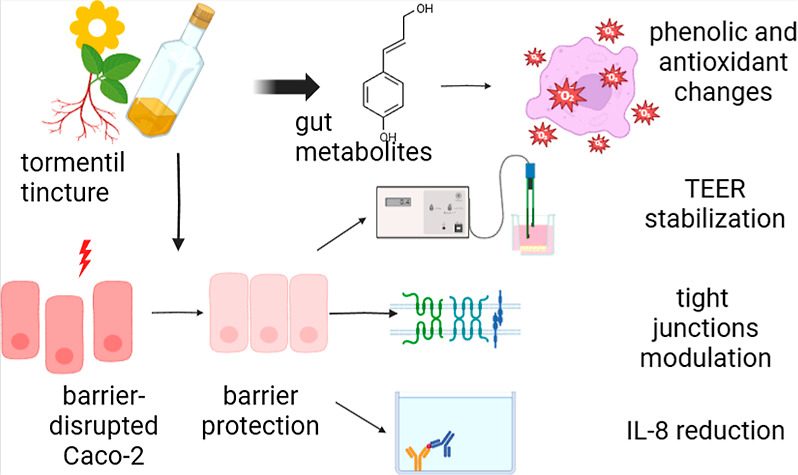

## 1 Introduction

In recent years, plant-derived specialized metabolites have emerged as valuable candidates for the development of functional foods, offering natural and sustainable means to strengthen intestinal health.^1–3^ Growing attention is directed toward their potential to mitigate conditions linked to impaired intestinal barrier function, a disorder increasingly recognized as a crucial factor in the pathophysiology of gastrointestinal and systemic diseases.^4,5^ Disruption of the epithelial barrier leads to increased intestinal permeability, facilitating the translocation of pathogens, toxins, and other harmful substances outside the gut lumen.^3^ This process promotes low-grade chronic inflammation and has been implicated in gastrointestinal and metabolic disorders, including irritable bowel syndrome, inflammatory bowel disease,^6^ obesity, diabetes.^7,8^

A pivotal element of barrier function is the network of tight junctions (TJs), multiprotein complexes composed of claudins, occludin, and zonula occludens proteins, collectively referred to as tight junction proteins (TJPs). These structures regulate paracellular transport and maintain polarity of the intestinal epithelium.^9,10^ Disruption or dysregulation of TJs results in a “leaky gut” syndrome, amplifying inflammatory responses and disease risk. Therefore, strategies that restore TJ integrity are considered crucial in therapies targeting intestinal permeability.^11^ Although pharmacological and dietary interventions may help manage inflammation-associated intestinal dysfunction, strategies specifically targeting restoration of epithelial barrier integrity remain insufficiently defined.^12,13^

Probiotics and prebiotics are widely studied, yet their efficacy varies significantly among individuals due to host- and microbiota-related factors. This development has promoted considerable interest in dietary interventions, with plant-derived functional food ingredients emerging as especially promising candidates.^14,15^ Plants provide a rich source of bioactive compounds, such as polyphenols, flavonoids, and tannins, which can modulate redox balance,^16^ regulate inflammatory pathways,^17^ and reinforce TJ integrity.^18^ Importantly, these compounds are metabolized by the gut microbiota into bioactive metabolites with modified absorption, bioavailability, and biological functions, offering additional beneficial properties.^19^ These compounds interact in a bidirectional manner, contributing to gut microbiota homeostasis, often through their beneficial effects on microbial diversity.^20^ Although plant-derived polyphenols are widely recognized as modulators of intestinal barrier function, their biological activity after gut microbiota-mediated transformation remains insufficiently understood. This knowledge gap is particularly relevant for tannin-rich extracts, such as tormentil rhizome ethanolic extract (TR-EtOH), whose major constituents undergo extensive microbial degradation before interacting with the intestinal epithelium. Therefore, it remains unclear whether the protective effects of TR-EtOH are driven mainly by native polyphenols or their microbiota-derived metabolites.

Tormentil rhizome ethanolic extract (TR-EtOH) occupies a distinctive place among these preparations. The rhizome of *Potentilla erecta* has been used for centuries in European traditional medicine for gastrointestinal ailments such as diarrhea and inflammation. Its phytochemical profile is dominated by hydrolyzable and condensed tannins, together with phenolic acids and flavonoids, which account for its strong antimicrobial, antioxidant, and anti-inflammatory properties.^21,22^ Today, tormentil tincture remains popular as a functional alcoholic beverage, particularly in Central and Eastern Europe, valued not only for its distinctive astringent taste but also for its health-promoting properties. Additionally, it has been applied as a natural preservative in brewing due to its antimicrobial activity.^23^ Upon ingestion, TR-EtOH polyphenols are transformed by the gut microbiota into metabolites with distinct bioactivity that may modify the extract’s protective effects, similar to other orally administered plant extracts.^24,25^

Taken together, plant-derived functional food ingredients, as demonstrated by tormentil rhizome extract, represent a sustainable and promising strategy to support intestinal barrier function. Understanding their biotransformation profile and biological activity is crucial for developing innovative dietary approaches aimed at preventing or mitigating disorders associated with impaired gut integrity.

The present study aimed to investigate the barrier-protective and anti-inflammatory effects of TR-EtOH and its gut microbiota-derived metabolites (TREMs, tormentil rhizome ethanolic extract metabolites) using a model of acute and prolonged intestinal epithelial injury induced by *Clostridioides difficile* toxins. This model was selected because *Clostridioides difficile* toxins directly disrupt tight junction organization and epithelial barrier function, providing a rapid and reproducible system for investigating barrier-protective interventions compared with conventional inflammatory models that often induce only gradual and variable changes in epithelial permeability. The study included assessment of transepithelial electrical resistance (TEER), expression and production of tight junction proteins and cytokine secretion, together with evaluation of how gut microbial metabolism modifies the phytochemical profile and antioxidant activity of the extract.

## 2 Materials and Methods

### 2.1 Preparation of TR-EtOH and Biosynthesis of TREMs

Tormentil rhizome (Kawon, Gostyń, Poland) was used to obtain the ethanolic extract and its gut microbiota-derived metabolite fractions, prepared as previously described by.^24^ In brief, TR-EtOH was produced via ultrasound-assisted extraction with an ethanol–water mixture (70%), followed by filtration, concentration, freeze-drying, and storage at 4 °C.^23^

Fecal fermentations were carried out using anonymized samples obtained from three healthy adult donors (D1–D3) aged 26–36 years. The study protocol was reviewed and approved by the Ethics Committee of the Medical University of Warsaw (approval no. AKBE/151/2021) and was conducted in accordance with the Declaration of Helsinki. All donors provided written informed consent prior to sample collection. The collected samples were anonymized before further processing and analysis. Prior to sample collection, donors followed a low-polyphenol diet for at least 3 days and had not used probiotic or prebiotic supplements for at least six months. All donors were healthy adults with no history of gastrointestinal disorders. Human fecal slurries were prepared in Brain Heart Infusion broth (BHI, Roth, Craponne, France) and incubated anaerobically in Bactron 300 anaerobic chamber (Shelton Scientific, Shelton, CT, USA) in the presence of the aqueous TR-EtOH stock (final concentration 2 mg/mL) for 24 h. Controls included only fecal slurries which were incubated separately with the medium. Postincubation, samples were centrifuged and extracted using solid-phase extraction with either 30% methanol (fractions TREM-D1-30, TREM-D2-30, TREM-D3-30) or 100% methanol (fractions TREM-D1-100, TREM-D2-100, TREM-D3-100). Coding system of obtained TREMs fraction is presented in Table 1. Fractions were then dried by solvent evaporation and freeze–drying. For all assays, TREMs were applied at relative concentrations corresponding to the amount of metabolites obtained from the parent extract during the fermentation process, allowing direct comparison between the biological activity of TR-EtOH and its gut microbiota-derived metabolite fractions. For clarity, these concentrations are hereafter referred to as concentrations equivalent to 1 mg/mL of TR-EtOH. Filtered metabolite fractions (TREMs) were stored until further chemical and biological evaluation.^24^

**Table 1:** Coding System of Tormentil Rhizome Ethanolic Extract Metabolites (TREMs) Derived from Three Donors

donor	fraction eluted with 30% MeOH	fraction eluted with 100% MeOH
D1	TREM-D1-30	TREM-D1-100
D2	TREM-D2-30	TREM-D2-100
D3	TREM-D3-30	TREM-D3-100

### 2.2 Chromatographic Analysis of TR-EtOH and TREMs

The chemical composition of TR-EtOH was qualitatively investigated using an ultrahigh-performance liquid chromatography–mass spectrometry system (UHPLC-MS; UHPLC-3000 RS, Dionex, Leipzig, Germany) coupled with a diode array detector (DAD) and an AmaZon SL ion trap mass spectrometer (Bruker Daltonik GmbH, Bremen, Germany) equipped with an electrospray ionization (ESI) source. Separation was performed on a Kinetex XB-C18 column (150 mm × 3.0 mm, 2.6 μm), using a gradient of water and acetonitrile, both containing 0.1% formic acid. UV spectra were recorded between 200 and 450 nm, and MS data were acquired in the *m*/*z* range 70–2200. This setup enabled identification of major constituents based on their retention times, UV absorption, and mass fragmentation patterns.

Quantitative analysis of the extract was carried out using external calibration with representative standards, including (−)-epicatechin, α-hederin (Sigma-Aldrich, Saint Louis, MO, USA), and 3,3′-di-*O*-methylellagic acid 4′-xylopyranoside (PhytoLab GmbH & Co.KG, Vestenbergsgreuth, Germany) and agrimoniin (isolated in-house). Calibration curves showed excellent linearity, while limits of detection and quantification were determined for each compound. Peak areas at specific wavelengths were integrated from DAD chromatograms and confirmed by MS, and compound abundance was expressed as a percentage of the total quantified content.

TREMs fractions were analyzed separately using a Vanquish UHPLC system coupled to an Orbitrap Exploris 120 mass spectrometer (Thermo Fisher Scientific, Austin, TX, USA). Separation was performed on a Kinetex XB-C18 column (150 × 2.1 mm, 1.7 μm) with a mobile phase consisting of water (A) and acetonitrile/water 4:1 (B), both containing 0.1% formic acid and 5 mM ammonium formate. A stepwise gradient from 1% to 100% B was applied, and the column temperature was maintained at 45 °C. The mass spectrometer operated in polarity-switching ESI mode with data-dependent MS/MS acquisition to enable structural annotation of metabolites. Experimental samples were analyzed both with and without exclusion lists generated from controls to maximize metabolite detection. Raw data were processed using Compound Discoverer 3.4 (Thermo Fisher Scientific) for peak alignment, annotation, and quantitative evaluation.^24^

### 2.3 Total Phenolic Content Assay

The total phenolic content (TPC) of the TR-EtOH and TREMs was determined using the Folin–Ciocalteu method with gallic acid (GA) as the calibration standard. A 10% (*v*/*v*) Folin–Ciocalteu reagent (Sigma-Aldrich) was prepared by diluting 10 mL of the commercial reagent with deionized water to a final volume of 100 mL. A 1 M Na_2_CO_3_ solution was obtained by dissolving 21.2 g of sodium carbonate (Sigma-Aldrich) in 200 mL of deionized water. The GA (Sigma-Aldrich) stock solution (2.00 mg/mL) was prepared in 50% methanol, and working standard solutions were obtained by appropriate dilutions in the same solvent to establishment of calibration curve in the range of 10–300 μg/mL. The TR-EtOH was dissolved in 50% methanol (Avantor, Gliwice, Poland) to a final concentration of 1 mg/mL, while TREMs were dissolved in 50% methanol at concentrations equivalent to 1 mg/mL of the original extract. For the assay, 40 μL of each sample, standard, or blank (50% methanol) was pipetted into a 96-well microplate, followed by the addition of 105 μL of 10% Folin–Ciocalteu reagent and 85 μL of 1 M Na_2_CO_3_. After thorough mixing, the plate was incubated for 15 min at 420 rpm on a plate shaker set at a 45° incline. Absorbance was measured at 765 nm using a Synergy 4 microplate reader (BioTek Instruments, Winooski, VT, USA). TPC was calculated from the gallic acid calibration curve and expressed as milligrams of gallic acid equivalents per gram of dry extract (mg GAE/g TR-EtOH). Measurements were conducted in three independent replicates, with results reported as mean ± standard deviation.^26^

### 2.4 DPPH Radical Scavenging Assay

The antioxidant activity of the tested samples was evaluated using the 2,2-diphenyl-1-picrylhydrazyl (DPPH) radical scavenging assay. A 0.02 mM solution of DPPH (Sigma-Aldrich) was prepared in methanol (Avantor). Stock solutions of the tested samples were prepared at a concentration of 1 mg/mL in 50% (*v*/*v*) ethanol (Avantor). Subsequently, working solutions were prepared by diluting the stock solutions with 50% (*v*/*v*) ethanol to obtain final concentrations of 10, 20, 50, 150, and 250 μg/mL. l-ascorbic acid (LA; Sigma-Aldrich) was used as a positive control. A stock solution (1 mg/mL in 50% ethanol) was prepared and diluted to obtain final concentrations of 1, 3.5, 7, 10, and 15 μg/mL. In a 96-well plate, 100 μL of each sample dilution or control solution was mixed with 100 μL of the DPPH solution. The reaction mixtures were incubated in the dark at room temperature for 30 min. After incubation, the absorbance was measured at 518 nm using a Synergy 4 microplate reader (BioTek Instruments). A mixture of methanol and DPPH served as the negative control, while methanol was used for baseline correction. The percentage of DPPH radical scavenging activity was calculated using the following formula

$$\%inhibition=100-\frac{\left(A_{sample}-A_{blindsample}\right)\times 100}{A_{control}}$$

The IC_50_ value, defined as the concentration of the sample required to scavenge 50% of DPPH radicals, was determined by plotting the percentage of inhibition against the logarithm of the sample concentration. Nonlinear regression analysis was performed using GraphPad Prism 9.0 (GraphPad Software, San Diego, CA, USA). Dose–response curves were used to calculate IC_50_ values for the tested samples and for ascorbic acid. All measurements were carried out in triplicate, and the results are presented as mean ± standard deviation.^26^

### 2.5 Preparation of TcdA/TcdB Mixture

To obtain the toxin-containing culture supernatant, *Clostridioides difficile* ribotype 078 spores were inoculated into BHI medium (Roth) supplemented with yeast extract (Sigma-Aldrich) and taurocholate (Sigma-Aldrich), and incubated at 37 °C for 7–14 days. After incubation, the culture was centrifuged at 10,000*g* for 10 min, and the supernatant was filtered through a 0.22 μm membrane to remove bacterial cells. The concentrations of toxins A and B (TcdA and TcdB), collectively referred to as *Clostridioides difficile* toxins (CD), were quantified using a commercial ELISA kit (gcBIOMICS GmbH, Bingen, Germany).^27^

### 2.6 Caco-2 Cell Culture

Caco-2 cells (ACC 169), obtained from the DSMZ (Braunschweig, Germany), were routinely cultured in plastic cell culture flasks (Greiner Bio-One, Kremsmünster, Austria) at 37 °C in a humidified atmosphere containing 5% CO_2_. The cells were grown in minimum essential medium eagle with Earle’s salts, l-glutamine and sodium bicarbonate (Sigma-Aldrich) supplemented with MEM nonessential amino acid solution (Sigma-Aldrich) and 20% of fetal bovine serum (Sigma-Aldrich). Cells were passaged at 70–80% confluence using 0.25% trypsin-EDTA. Cells between passages 7 and 28 were used for all experiments. All assays with Caco-2 cells were performed in at least three biologically independent replicates, each tested in technical triplicates.^23^

### 2.7 Tight Junction Disruption Model Establishment and TEER Measurement

Caco-2 cells were seeded at a density of 6 × 10^5^ cells per well on Transwell inserts (Greiner Bio-One, Kremsmünster, Austria) and cultured for 21 days to achieve full differentiation and confluence. Cell number and viability were determined using an EVE Plus automated cell counter (NanoEnTek Inc., Seoul, Republic of Korea). The culture medium was refreshed every 3 days. To establish the tight junction disruption model, differentiated monolayers were incubated for 24 h with various agents, including lipopolysaccharide (LPS; *E. coli*, Merck, Darmstadt, Germany), interferon-γ (IFN-γ), tumor necrosis factor (TNF-α), mefenamic acid (MA), and deoxynivalenol (DON; all Sigma-Aldrich). Transepithelial electrical resistance (TEER) was measured at selected time points during this 24 h incubation using an EVOM2 voltmeter (World Precision Instruments, Sarasota, FL, USA) or Cellzcope device (nanoAnalytics, Münster, Germany) to monitor changes in barrier integrity and to identify appropriate conditions for subsequent experiments. All experiments were performed using at least three independent biological replicates.

### 2.8 Intestinal Barrier Integrity

On 21 day differentiated Caco-2 monolayers, the medium was replaced with fresh medium containing the tested samples (stock solutions in DMSO; Thermo Fisher Scientific, diluted in culture medium). Sodium butyrate (NaB, Merck) at concentration 5 mM was used as a positive control. Medium with 0.5% DMSO served as the nontreated control (NTC). TEER measurements were performed at selected time points during the 24 h incubation with test samples using the EVOM2 voltmeter to evaluate their effects on barrier integrity. Following this, the TcdA/TcdB mixture was added at a final concentration of 2.5 and 0.8 ng/mL, respectively, and cells were incubated for an additional 6 h. TEER was measured hourly during the 6 h toxins mixture exposure using the same device to assess TJ disruption over time. Cells treated only with the toxins mixture served as a treated control (CD).^27,28^ Barrier integrity experiments were performed using at least three independent biological replicates.

### 2.9 Quantitative Real-Time PCR for TJP Gene Expression

For quantitative real-time PCR, Caco-2 cells were cultured and treated under the same conditions, using identical controls and test samples as in the barrier integrity assays. Following 24 h preincubation with the test samples and a subsequent 6 h coincubation with both the test samples and the TcdA/TcdB mixture (2.5 and 0.8 ng/mL, respectively), cells were washed with ice-cold PBS, detached using a sterile cell scraper, and transferred to 1.5 mL microcentrifuge tubes. Total RNA was extracted using the NucleoSpin RNA II kit (Macherey–Nagel, Düren, Germany) in accordance with the manufacturer’s protocol. RNA concentration and integrity were verified using the RNA 6000 Nano Kit and Agilent 2100 Bioanalyzer system (Agilent Technologies, Waldbronn, Germany). Complementary DNA (cDNA) synthesis was carried out with the SuperScript III First-Strand Synthesis System (Thermo Fisher Scientific) in a SureCycler 8800 thermal cycler (Agilent Technologies). Quantitative real-time PCR was conducted using the Brilliant II SYBR Green QPCR Master Mix with Low ROX on an Aria Mx Real-Time PCR system (Agilent Technologies). The qPCR reaction was performed with an initial denaturation at 95 °C for 10 min, followed by 40 amplification cycles consisting of denaturation at 95 °C for 30 s, annealing at 60 °C for 30 s for β-actin (ACTB), TATA-box binding protein (TBP), occludin (OCLN), claudin-4 (CLDN-4) and zonula occludens-1 (ZO-1) or 58 °C for 30 s (for claudin-2, CLDN2), and elongation at 72 °C for 30 s. Subsequently, a melting curve analysis was carried out (95 °C for 30 s, 60 °C for 30 s, 95 °C for 30 s), followed by a final cooling step at 25 °C for 1 min. The relative expression levels of TJ-related genes were evaluated, including CLDN2, CLDN4, OCLN, and ZO-1. For normalization, ACTB and TBP were initially evaluated as reference genes. However, as ACTB showed more stable expression across experimental conditions, final normalization was performed using ACTB only. Results were expressed as fold change relative to control cells treated with medium with 0.5% DMSO. Primer sequences, along with their corresponding annealing temperatures and product lengths, are provided in Supporting Information in Table S1.^28^ Gene expression experiments were performed using at least three independent biological replicates.

### 2.10 Western Blot Analysis of Tight Junction Proteins

For Western blot analysis, Caco-2 cells were cultured and treated under the same conditions, using identical controls and test samples as in the barrier integrity assays. Following 24 h preincubation with the test samples and a subsequent 6 h coincubation with both the test samples and the TcdA/TcdB mixture (2.5 and 0.8 ng/mL, respectively), cells were rinsed twice with ice-cold PBS and lysed using cOmplete Lysis-M buffer (Roche, Basel, Switzerland) supplemented with protease and phosphatase inhibitors (Roche). Lysates were centrifuged at 14,000*g* for 15 min at 4 °C, and the supernatants were collected. Protein concentration was determined using the Quick Start Bradford Protein Assay kit (Bio-Rad, Hercules, CA, USA) according to the manufacturer’s instructions. Equal amounts of protein samples were mixed with 4× Laemmli Sample Buffer (Bio-Rad), boiled, and stored at −20 °C until analysis. Proteins were separated by SDS-PAGE, using 12.5% polyacrylamide gels for CLDN-2, CLDN-4, and OCLN, and 8% gels for ZO-1. For claudins, electrophoresis started at 80 V for 30 min, then was increased to 100 V. Occludin was separated at 200 V throughout, while ZO-1 gels ran initially at 50 V until proteins entered the gel, followed by 150 V. Following electrophoresis, proteins were transferred onto nitrocellulose membranes (0.45 μm, Bio-Rad). Membranes were blocked in Tris-buffered saline (TBS, Promega, Madison, WI, USA) containing 3% skim milk (Merck). Transfer conditions varied: 250 mA for 2 h for occludin, 200 mA for 50 min for claudins, and 250 mA for 3 h for ZO-1. Membranes were then incubated with primary rabbit monoclonal antibodies against CLDN-2 (Cell Signaling Technology, Danvers, MA, USA, Cat. No. 48120, clone E1H9O, 1:1000), CLDN-4 (Cell Signaling Technology, Cat. No. 94478, clone F8I6W, 1:1000), OCLN (Cell Signaling Technology, Cat. No. 91131, clone E6B4R, 1:1000), ZO-1 (Cell Signaling Technology, Cat. No. 13663, clone D6L1E, 1:1000), and β-actin as a loading control (Cell Signaling Technology, Cat. No. 4970, clone 13 × 10^5^, 1:1000), either for 2 h at room temperature (β-actin) or overnight at 4 °C. After washing with 3% skim milk TBS, membranes were incubated with horseradish peroxidase (HRP)-conjugated secondary antibodies (Cell Signaling Technology, Cat. No. 7074, 1:2000) for 1 h at room temperature, followed by additional washes in skim milk TBS and TBS with 0.1% Tween-20 (Merck). Protein bands were visualized using SuperSignal West Pico PLUS chemiluminescent substrate (Thermo Fisher Scientific) and imaged on a Bio-Rad ChemiDoc MP system (Hercules, CA, USA) under nonsaturating exposure conditions. Band intensities were quantified using Image Lab software (Bio-Rad, Hercules, CA, USA) and normalized to β-actin. Results were expressed as fold change relative to control cells treated with medium with 0.5% DMSO.^29^ Western blot experiments were performed using at least three independent biological replicates.

### 2.11 Cytokine Quantification by Cytometric Bead Array and ELISA

For cytokine quantification, Caco-2 cells were seeded into 24-well plates at a density of 150,000 cells/well. Upon reaching full confluence, the culture medium was replaced with fresh medium containing TR-EtOH or TREMs at appropriate concentrations. Stock solutions were prepared in DMSO and diluted in medium to achieve the desired final concentrations. Dexamethasone at concentration 20 μM (Dex, Sigma-Aldrich) was selected as a positive anti-inflammatory control based on previously published studies demonstrating effective modulation of inflammatory responses in intestinal epithelial cell models at comparable concentration ranges.^30^ As a nontreated control, cells were incubated with medium containing 0.5% DMSO. After 24 h of preincubation with the test samples, the TcdA/TcdB mixture was added to a final concentration of 2.5 and 0.8 ng/mL, respectively, while cells treated only with toxins served as the toxin-treated control (CD). Following an additional 6 h incubation, culture supernatants were collected. Cytokine levels (IL-12p70, TNF-α, IL-10, IL-6 and IL-1β) were quantified primarily using a Human Inflammatory Cytokine Cytometric Bead Array (CBA) kit combined with flow cytometry (BD FACSCelesta), while IL-8 concentration was determined separately using a BD OptEIA Human IL-8 ELISA kit (BD Biosciences, Franklin Lakes, NJ, USA), according to the manufacturers’ instructions. ELISA absorbance was measured using a Synergy 4 microplate reader (BioTek). Cytokine quantification experiments were performed using at least three independent biological replicates.

### 2.12 Statistical Analysis

Calculations were performed using Microsoft Excel (Microsoft Office 365, Microsoft Corporation, Redmond, WA, USA). Statistical analyses to determine significant differences between tested samples and the control were conducted using one-way analysis of variance (ANOVA), followed by Dunnett’s post hoc test. Correlation analysis was performed using Spearman’s correlation test. A *p*-value of less than 0.05 was considered statistically significant. All statistical analyses were carried out using Statistica 10.0 software (StatSoft Inc., Tulsa, OK, USA).

## 3 Results and Discussion

### 3.1 Phytochemical Characterization of TR-EtOH and TREMs

Based on previous detailed phytochemical investigations of TR-EtOH, including both qualitative and quantitative analyses,^23^ as well as separate studies on its gut-derived metabolites,^24^ the present study provides a summarized and illustrative overview of its composition to support interpretation of the biological results obtained for TR-EtOH (Table 2) and its gut-derived metabolites (Table 3). This analysis is intended only as a concise representation, while the full structural characterization, quantitative data, and detailed UHPLC–MS chromatographic profiles of the extract’s constituents have been reported elsewhere and are provided in Supporting Information (Tables S2 and S3).

**Table 2:** Summary of Compound Classes Identified in the Tormentil Rhizome Ethanolic Extract by LC–MS/MS, Including Representative Compounds, Diagnostic Fragment Ions Recorded in Negative Mode, and Quantitative Content^a^

phytochemical class	detected compounds (*m*/*z*)	content
flavan-3-ols and procyanidins	catechin (289);	dominant
	catechin-*O*-hexoside (451);	
	catechin-(epi)afzelechin dimers (561);	
	procyanidin dimers (B-type) (577);	
	procyanidin trimers (C-type) (865);	
	procyanidin tetramers (576);	
	procyanidin pentamers (720)	
ellagitannins and ellagic acid derivatives	ellagic acid (301);	minor
	ellagic acid *O*-pentoside (433);	
	ellagic acid *O*-hexoside (463);	
	methylellagic acid *O*-hexoside (477);	
	galloyl-ellagic acid *O*-hexoside (615);	
	methylellagic acid *O*-pentosides (447);	
	methylellagic acid *O*-glucuronide (491);	
	agrimoniin (934)	
flavonoids and related compounds	phlorizin (435);	low
	flavonoid derivatives (575, 605)	
phenolic acids	protocatechuic acid *O*-hexoside (315);	not determined
	gallic acid derivatives (377)	
triterpenoids	tormentic acid isomers (487);	substantial
	tormentic acid *O*-glycosides (695);	
	ursane-type triterpenoid glycosides (693, 695);	
	myrianthic acid (503);	
	cecropiacic acid (517);	
	triterpenoid hexosides (679)	
other constituents	several unidentified compounds (441, 511, 507, 493, 711, 605)	not determined

a Both qualitative identification and quantitative data were based on previously published LC–MS/MS analyses.^23^

**Table 3:** Gut Microbiota-Derived Metabolites and Native Constituents of Tormentil Rhizome Ethanolic Extract Detected in Tormentil Rhizome Ethanolic Extract Metabolites Fractions by LC–MS, Including Semi-quantitative Abundance Based on Peak Area Ratios in Negative Mode^a^

identification (*m*/*z*)	TREM-D1–30	TREM-D1–100	TREM-D2–30	TREM-D2–100	TREM-D3–30	TREM-D3–100
microbiota-derived flavan-3-ol metabolites (postbiotics)						
catechin derivative (291)	+	+	++	++	+++	+++
procyanidin B/C derivative (354)	+	+	++	++	+++	+++
procyanidin B/C derivative (369)	++	+++	++	+++	+	+
catechin derivative (396)	+	+++	++	+++	+	++
procyanidin B/C derivative (396)	+	+++	++	+++	+	++
catechin derivative (167)	+	+++	+	+++	++	++
catechin derivative (371)	+	++	+	+++	++	+++
procyanidin B/C derivative (398)	+	++	+	+++	++	+++
catechin derivative (291)	+	++	+	+++	++	+++
catechin derivative (315)	+	+++	++	+++	+	++
catechin derivative (271)	++	+++	+	+++	+	++
catechin derivative (273	+	+++	++	+++	+	++
catechin derivative (285)	++	+++	++	+++	+	+
catechin derivative (209)	+	+++	+	++	++	+++
catechin derivative (255)	++	+++	+	++	+	+++
native flavan-3-ols directly derived from the extract (nontransformed)						
procyanidin dimer type B isomer I (577)	++	–	++	–	+++	–
catechin (289)	++	–	+	–	+++	–
procyanidin trimer type C isomer II (865)	++	–	+	–	+++	–
native ellagic acid derivatives directly derived from the extract (nontransformed)						
ellagic acid (301)	–	++	–	+	–	+++
methylellagic acid *O*-pentoside isomer II (447)	–	+++	–	++	–	+
triterpenoids and their glycosides directly derived from the extract (nontransformed)						
tormentic acid *O*-hexoside (695)	–	+	–	++	–	+++
tetrahydroxy-methoxy ursane hexoside (695)	–	+	–	++	–	+++
tormentic acid *O*-hexoside isomer II (695)	–	++	–	+	–	+++
trihydroxy ursane hexoside (695)	–	+	–	++	–	+++
dihydroxy-oxo ursane hexoside (693)	–	+	–	++	–	+++
dihydroxy-oxo ursane hexoside isomer (693)	–	++	–	+++	–	+
undefined triterpenoid hexoside (679)	–	+	–	++	–	+++
cecropiacic acid (517)	–	+	–	+++	–	++
tormentic acid isomer I (487)	–	+	–	++	–	+++
unknown compounds directly derived from the extract (nontransformed)						
unknown (507)	–	++	–	+++	–	+
unknown (493)	–	+++	–	+	–	++
unknown (493)	–	++	–	+	–	+++
unknown (507)	–	+	–	++	–	+++

a Semi-quantitative abundance is indicated as – (not detected), + (ow), + + (moderate), + ++ (high). Identification and relative abundance are based on previously published LC–MS/MS analyses.^24^

The extract was dominated by flavan-3-ols and their oligomeric procyanidins, including catechin, catechin-*O*-hexoside, catechin-(epi)­afzelechin dimers, B-type procyanidin dimers, C-type procyanidin trimers, as well as tetramers and pentamers. Ellagitannins and ellagic acid derivatives were also detected, such as ellagic acid, ellagic acid *O*-pentoside, *O*-hexoside, methylellagic acid *O*-hexoside, galloyl-ellagic acid *O*-hexoside, methylellagic acid *O*-pentosides, methylellagic acid *O*-glucuronide, and agrimoniin. Flavonoids, including phlorizin and other flavonoid derivatives, were present at low levels. Phenolic acids, such as protocatechuic acid *O*-hexoside and gallic acid derivatives, were also identified, although their relative contribution was not determined. Triterpenoids constituted a substantial fraction of the extract and included tormentic acid isomers, tormentic acid *O*-glycosides, ursane-type triterpenoid glycosides, myrianthic and cecropiacic acids, as well as other triterpenoid hexosides. Additionally, several unidentified compounds with distinct m/z values (e.g., 441, 511, 507, 493, 711, 605) were observed, reflecting the chemical diversity of the extract.

This compositional summary highlights that TR-EtOH contains multiple bioactive classes, including condensed and hydrolyzable tannins, flavonoids, phenolic acids, and triterpenoids, which may contribute to the biological effects explored in subsequent experiments.

The detailed phytochemical analysis of the TREMs fraction showed that several native extract constituents remained untransformed in the postincubation mixtures. Catechins and procyanidin oligomers, including catechin, procyanidin dimers, and trimers, were primarily detected in the 30% methanol fractions, whereas ellagic acid derivatives (e.g., ellagic acid, methylellagic acid *O*-pentoside) and glycosylated triterpenoids (e.g., tormentic acid *O*-hexoside and related ursane-type derivatives) were enriched in the 100% methanol fractions. Additionally, several unidentified metabolites (m/z 493 and 507) were consistently observed across all donors. While the qualitative metabolite profiles were similar among donors, quantitative differences were apparent, reflecting interindividual variability in gut microbiota composition and metabolic activity. In particular, donor 3 generally exhibited higher signal intensities for both catechin-type and triterpenoid derivatives. Overall, new compounds detected in the postincubation mixtures represented potential gut microbiota-derived metabolites, whereas detectable changes were observed primarily for flavan-3-ols and their oligomers under the experimental conditions used.

This summarized compositional overview demonstrates that flavan-3-ols and procyanidins are the primary constituents metabolized by the gut microbiota, and that both solvent polarity and interindividual microbiota variability influence the distribution and relative abundance of the resulting metabolites. The present data serve as a concise reference for understanding the biological effects of TREMs described in the following sections.

### 3.2 Alterations in Phenolic Content and Antioxidant Activity of TR-EtOH after Gut Microbiota Metabolism and Their Correlation

Table 4 and Figure 1 illustrate the total phenolic content and antioxidant activity of the original tormentil rhizome extract and its metabolite fractions. As shown in the table, TR-EtOH demonstrated the highest TPC (0.287 ± 0.0047 g GAE/g DW) and the strongest antioxidant activity (IC_50_ 14.39 ± 0.78 μg/mL), while the metabolites fractions exhibited markedly lower phenolic content and weaker antioxidant potential. Among the TREMs, TREM-D1-30, TREM-D2-30 and TREM-D3-30 showed relatively high TPC (0.099 ± 0.0030, 0.111 ± 0.0044 and 0.125 ± 0.0074 g GAE/g DW, respectively), aligned with favorable antioxidant profile (IC_50_ 42.56 ± 6.05, 39.87 ± 2.98 and 31.02 ± 2.65 μg/mL, respectively). In contrast, TREM-D1-100, TREM-D2-100 and TREM-D3-100, characterized by the lowest phenolic contents (0.010 ± 0.0022, 0.021 ± 0.013 and 0.014 ± 0.009, respectively) were significantly less active (IC_50_ 125.53 ± 8.80, 364.30 ± 79.05 and 96.14 ± 3.51 μg/mL, respectively). The bar chart (Figure 1) confirms these observations by displaying the dose-dependent inhibition of DPPH radicals by TR-EtOH at concentrations ranging from 7.8 to 1000 μg/mL (61.3–92.2%). Notably, TR-EtOH and ascorbic acid (positive control) exerted pronounced inhibitory activity even at the lowest tested concentrations, whereas the TREMs displayed weaker effects. For example, at 62.5 μg/mL, both TR-EtOH and LA achieved nearly complete inhibition (>90%), while most TREMs required much higher concentrations to reach similar levels. The TREM-D1-100, TREM-D2-100 and TREM-D3-100 in particular demonstrated poor radical scavenging activity across all tested concentrations (12.9–95.7, 5.7–91.9 and 11.3–94.9%, respectively). Remaining metabolites, TREM-D1-30, TREM-D2-30, and TREM-D3-30 exhibited moderate inhibitory activity (26.0–95.8, 25.8–95.3 and 27.6–95.8%, respectively). A strong negative correlation was observed between TPC and IC_50_ values (Spearman’s R = −0.89, p = 0.0068).

**Table 4:**
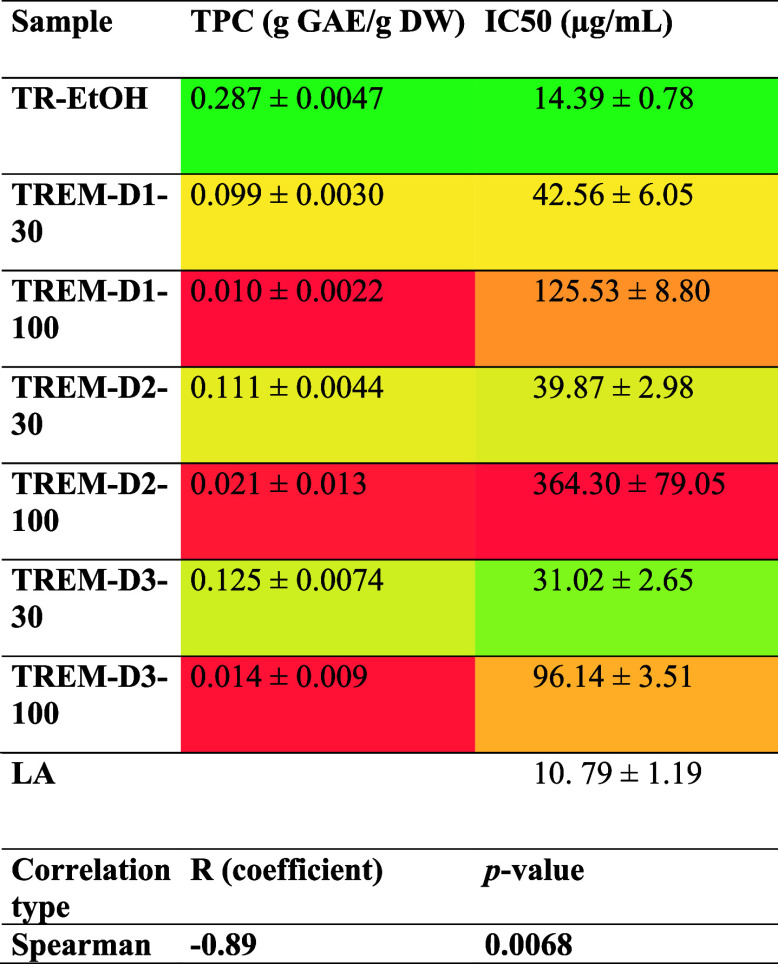
Total Phenolic Content (TPC) Expressed as Grams of Gallic Acid Equivalents Per Gram of Dry Weight (g GAE/g DW) and Antioxidant Activity (IC_50_ Values in μg/mL) of TR-EtOH and TREMs. l-ascorbic Acid (LA) was Used as Positive Control in Antioxidant Activity. The Table Also Presents the Spearman Correlation Coefficient (*R*) and *p*-Value between TPC and IC_50_ Values. Color coding: In the TPC Column, Green Indicates High Phenolic Content and Red Indicates Low Phenolic Content. In the IC_50_ Column, Green Indicates High Antioxidant Activity (Low IC_50_), and Red Indicates Low Antioxidant Activity (High IC_50_)

**Figure 1:**
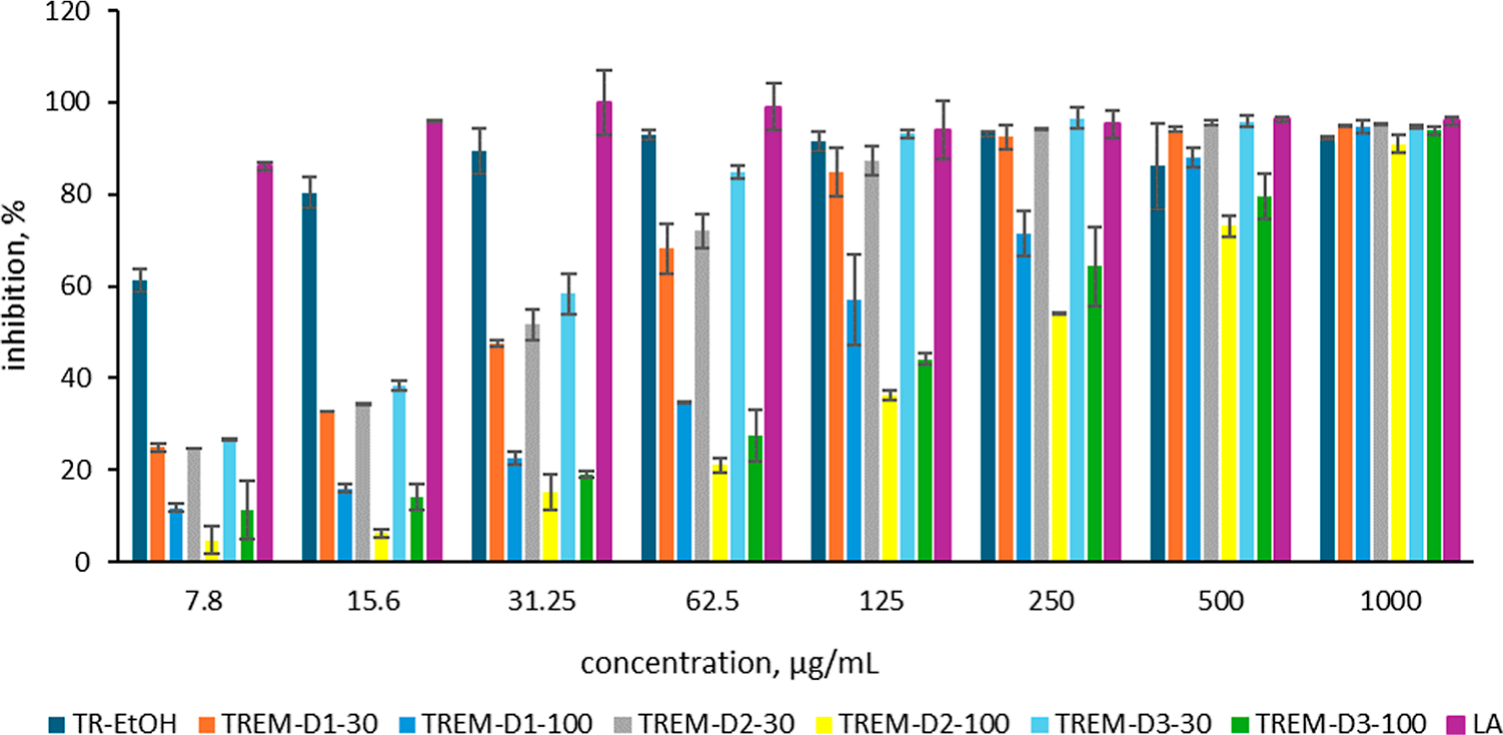
DPPH radical scavenging activity of TR-EtOH, TREMs and l-ascorbic acid (LA) at concentrations of 7.8–1000 μg/mL (for TREMs, concentrations were expressed as TR-EtOH equivalents). LA was used as a positive control. Results are expressed as percentage inhibition of the DPPH radical. Data are presented as mean ± SD (n = 3).

The high total phenolic content observed for TR-EtOH is consistent with our previously published UHPLC-MS analyses, which revealed a rich phytochemical profile dominated by procyanidins, ellagic acid derivatives, tormentic acid derivatives, and other triterpenoids. Our previously published metabolite analyses further demonstrated that TR-EtOH is metabolized by the gut microbiota into catechin oligomers derivatives.^24^ These compounds are known for their strong antioxidant potential and contribute significantly to the low IC_50_ value recorded in the DPPH assay. Procyanidins, as polymeric flavan-3-ols, exhibit potent radical-scavenging properties due to their multiple hydroxyl groups and capacity to stabilize reactive oxygen species. Similarly, ellagic acid derivatives have demonstrated robust antioxidant and anti-inflammatory properties, while triterpenoids such as tormentic acid derivatives may also contribute to the observed antioxidant activity.

These findings demonstrate that gut microbiota metabolism of TR-EtOH significantly reduces its total phenolic content and antioxidant capacity, as confirmed by the lower TPC and higher IC_50_ values observed for the metabolite fractions. This decrease is likely due to microbial degradation or transformation of larger polyphenolic structures, particularly procyanidins, into smaller, less active metabolites, as we have previously reported.^24^ Nonetheless, some individual fractions, such as TREM-D1-30 and TREM-D2-30, still retained moderate antioxidant activity, suggesting that certain microbiota metabolites may retain antioxidant activity despite extensive biotransformation. The observed correlation between phenolic content and antioxidant potential suggests that polyphenols contribute substantially to the antioxidant properties of the extract. Changes induced by gut microbiota in chemical composition and antioxidant potential may influence the biological properties of TR-EtOH and its metabolite fractions, particularly in the context of intestinal health.^31^ Because oxidative stress is one of the factors implicated in intestinal barrier dysfunction,^32,33^ the effects of TR-EtOH and TREMs on epithelial barrier integrity and inflammatory responses were subsequently investigated using the Caco-2 model.

### 3.3 Selection of the Intestinal Barrier Disruption Model

To establish an in vitro model of TJ disruption in intestinal epithelial cells, a range of well-known barrier-damaging agents were initially tested on differentiated Caco-2 monolayers. These included the mycotoxin deoxynivalenol, pro-inflammatory cytokines (TNF-α, IFN-γ), bacterial components such as lipopolysaccharide, and the nonsteroidal anti-inflammatory drug mefenamic acid, applied to either the apical or basolateral side of the monolayers or both (Supporting Information, Figures S1–S4). All mentioned compounds are known to impair epithelial barrier function through different molecular mechanisms: DON disrupts nonselectively protein synthesis and activates MAPK (mitogen-activated protein kinase) pathways;^34^ cytokines alter TJ protein expression trough NF-κB (nuclear factor kappa B) and JAK-STAT (Janus kinase-signal transducer and activator of transcription) signaling;^35,36^ LPS activates TLR4-mediated inflammation;^37^ and MA inhibits prostaglandin synthesis and induces oxidative stress.^38,39^ Despite these established effects, none of these factors, applied individually or in combination, led to a strong and permanent decrease in TEER under the experimental conditions used in the present study. Although previous studies have reported TEER reduction following exposure to LPS or pro-inflammatory cytokines, the magnitude of the response depends strongly on experimental conditions, including cell differentiation status, exposure time, and stimulus concentration. This observation may reflect the gradual nature of their action and the need for longer exposure times in differentiated cells.

Given these limitations, to better suit the aims of this study, an alternative model of TJ disruption was established using *Clostridioides difficile* toxins A and B, which offer more rapid and permanent effects.^27^ Importantly, this approach was not intended to reflect *C. difficile* infection, but rather to provide a robust and reproducible experimental system for inducing tight junction disruption and investigating barrier-protective interventions in intestinal epithelial cells. *C. difficile* toxins are well characterized for their direct and potent effects on epithelial junctional complexes. TcdA and TcdB are glucosyltransferases that inactivate small Rho GTPases (Rho, Rac, Cdc42), leading to depolymerization of the actin cytoskeleton and subsequent disassembly of TJ proteins.^40,41^ Studies have shown rapid redistribution or degradation of key junctional components, including ZO-1, occludin, and claudin-1, within a few hours of exposure in Caco-2 and HT-29 cells.^42,43^

To determine the optimal conditions for TJ disruption, the TcdA/TcdB mixture was applied to Caco-2 monolayers at various concentrations (TcdA 0.5–35.0 ng/mL, TcdB 0.2–11.5 ng/mL), and TEER was monitored over 24 h. As shown in the upper part of Figure 2, all tested concentrations induced a time- and dose-dependent decrease in TEER. Higher doses (TcdA 20.0 and 35.0 ng/mL, TcdB 6.6 and 11.5 ng/mL) triggered rapid and deep barrier disruption, whereas 2.5 ng/mL (TcdA) and 0.8 ng/mL (TcdB) caused a moderate but reproducible decrease in TEER within 6–8 h (zoomed-in view, lower part of Figure 2). For all concentrations, prolonged incubation led to a critical decrease in TEER (below 40%). Based on these findings, 2.5 ng/mL (TcdA) and 0.8 ng/mL (TcdB) were selected as the optimal concentration to model tight junction disruption, and a 6 h incubation was chosen as the optimal time point for subsequent testing of protective or restorative interventions in intestinal epithelial cells. For the selected concentration of *C. difficile* toxins (TcDA and TcdB 2.5 and 0.8 ng/mL, respectively) and exposure time (6 h), cell viability was assessed using the MTT assay (Supporting Information, Figure S5). The results showed that metabolic activity in toxins-treated cells was slightly elevated compared to the nontreated control (124.4 ± 5.1% vs 100.0 ± 6.7%, respectively), suggesting that the observed TEER reduction was not due to cytotoxicity. This mild increase in MTT assay may reflect a compensatory cellular response to early stress rather than enhanced viability.^44,45^ These findings support the interpretation that the toxins primarily caused functional disruption of TJ, rather than loss of cell viability or monolayer integrity.

**Figure 2:**
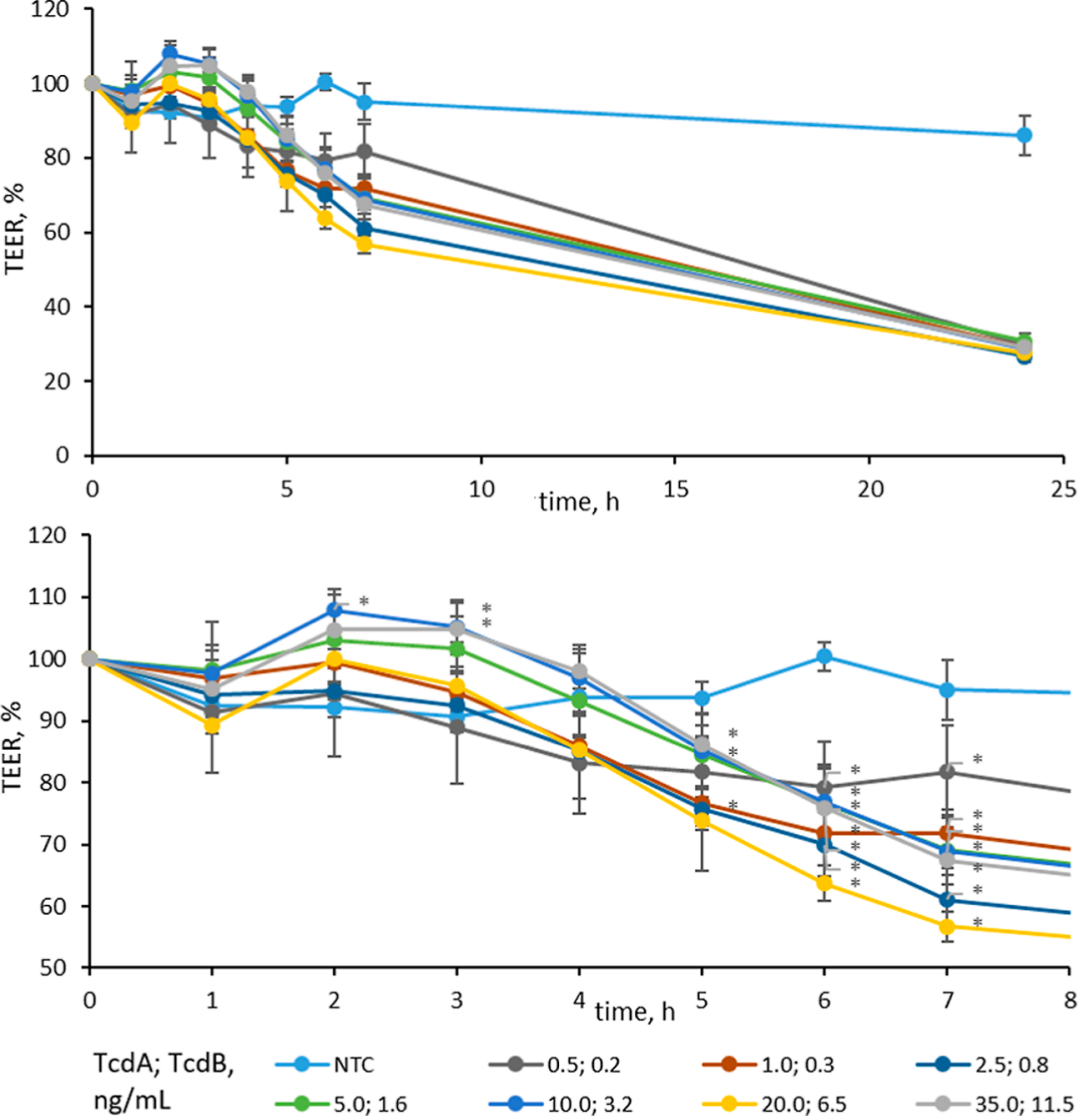
Effect of the TcdA/TcdB mixture (0.5–35 ng/mL) on TEER value of Caco-2 cell monolayers. Toxins were added at time 0 h. Lower graph present a zoomed-in view of the 1–8 h time frame. TEER values are expressed as % of baseline. Results are presented as mean ± SD (n = 3). Statistical significance was calculated using Dunnett’s post hoc test at *p* ≤ 0.05 versus nontreated control (NTC, *) or versus toxin-stimulated control (CD, #).

To determine the most effective timing of application, the protective and restorative effects of TR-EtOH (0.5–1 mg/mL) were evaluated in relation to exposure to *C. difficile* toxins (2.5 and 0.8 ng/mL for TcdA and TcdB, respectively). Caco-2 cells were treated with TR-EtOH using three different schemes: preincubation (TR-EtOH added 24 h before toxin exposure), coincubation (TR-EtOH and toxins added simultaneously), and postincubation (TR-EtOH added 6 h after toxin exposure) (Figure 3). The concentration range of TR-EtOH was selected based on previously reported cytotoxicity assays.

**Figure 3:**
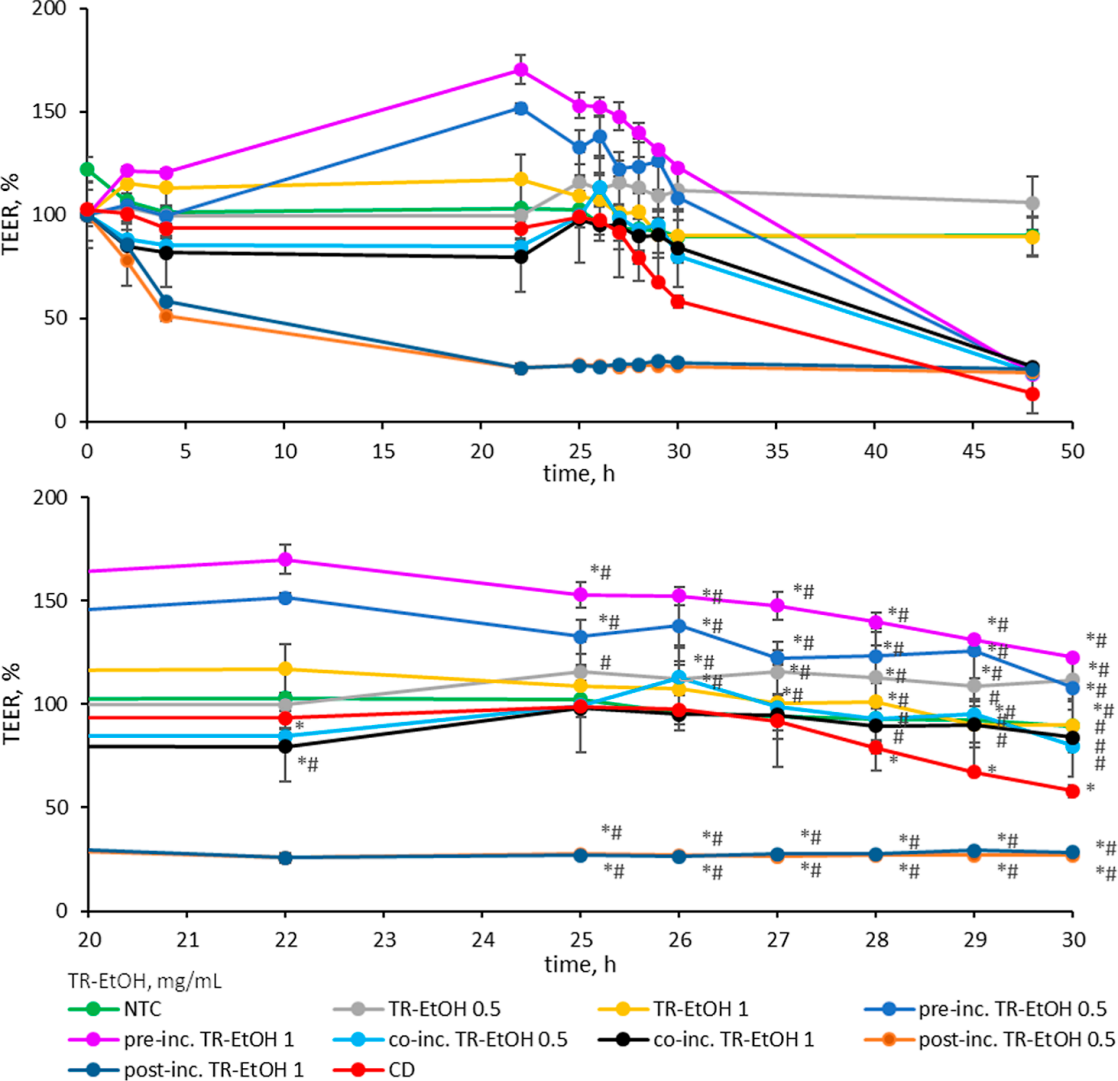
Effect of TR-EtOH (0.5–1 mg/mL) on TEER value of Caco-2 monolayers before (preinc.), during (coinc.), or after (postinc.) exposure to the TcdA/TcdB mixture (2.5 and 0.8 ng/mL, respectively). CD was added at 0 h (postinc.) or 24 h (preinc. and coinc.). Lower graph present a zoomed-in view of the 22–30 h time frame. TEER values are expressed as % of baseline. Results are presented as mean ± SD (n = 3). Statistical significance was calculated using Dunnett’s post hoc test at *p* ≤ 0.05 versus nontreated control (NTC, *) or toxin-stimulated control (CD, #).

Preincubation with TR-EtOH (0.5 and 1 mg/mL) significantly increased TEER at 6 h after toxins addition (108.1 ± 0.3% and 122.7 ± 1.5% for 0.5 and 1 mg/mL, respectively), compared to the toxins-treated control (58.0 ± 2.9%). Similarly, coincubation with the extract also increased TEER values at the same time point (79.9 ± 1.1% and 84.1 ± 18.9% for 0.5 and 1 mg/mL, respectively), relative to the toxins-treated control. In contrast, the addition of TR-EtOH after toxin exposure did not improve TEER at 6 h (26.8 ± 0.6% and 29.1 ± 2.1% for 0.5 and 1 mg/mL, respectively), with values remaining low, indicating persistent disintegration of the monolayer. Additionally, treatment only with TR-EtOH (without toxins), at both tested concentrations, did not affect TEER values, confirming that the extract does not alter stable, differentiated and undamaged monolayer integrity.

Presented studies showed that the timing of TR-EtOH applications played a crucial role in its ability to modulate epithelial barrier integrity. Preincubation with TR-EtOH exerted the strongest protective effect, and maintained barrier integrity during toxins exposure. Notably, TEER values exceeding 100% were observed during the preincubation period, particularly at the higher extract concentration. Since TEER reflects not only tight junction integrity but also other factors affecting epithelial electrical resistance, including ion transport, these elevated values should be interpreted with caution. Further studies would be required to determine the precise mechanisms underlying this effect. Co-incubation with TR-EtOH also showed a barrier-stabilizing effect, though less effectively. Postincubation with TR-EtOH did not restore TEER, suggesting that the extract’s activity is primarily preventive rather than regenerative. Since the aim of this study was to investigate the preventive potential of TR-EtOH in protecting epithelial barrier function, the preincubation protocol was selected for all subsequent experiments. This approach aligns with the concept of using plant-derived compounds not as therapeutic agents after damage has taken place, but as functional ingredients that help maintain epithelial homeostasis and reduce the risk of barrier dysfunction under stress conditions.

### 3.4 Effects of TR-EtOH and TREMs on Tight Junction Integrity

#### 3.4.1 Monolayer Stability Assessed by TEER Measurement

To further evaluate the barrier-stabilizing properties of tormentil extract under preventive conditions, Caco-2 monolayers were preincubated with TR-EtOH (0.25–1 mg/mL) for 24 h before to *C. difficile* toxins A and B addition (2.5 and 0.8 ng/mL, respectively). Sodium butyrate (5 mM), a short-chain fatty acid with well-documented epithelial barrier-enhancing effects, was used as a positive control.^46^ As shown in Figure 4, TEER remained stable or increased during the 24 h preincubation with TR-EtOH. After addition of toxins, cells pretreated with TR-EtOH exhibited a significantly slower decrease in TEER after 6 h of toxins exposure (116.4 ± 12.2, 105.7 ± 12.7 and 89.4 ± 9.4% for 0.25–1 mg/mL, respectively), compared to toxins-treated control (CD, 69.4 ± 5.6%). Notably, concentrations 0.25 and 0.5 mg/mL showed the most pronounced protective effect, even exceeding the efficacy of NaB at several time points of toxins exposure (0–6 h for 0.5 mg/mL and 4–6 h for 0.25 mg/mL, zoomed-in view in lower part of Figure 4). In contrast, the highest concentration (1 mg/mL) had only modest effects on TEER protection, indicating a nonlinear dose–response relationship. This finding suggests that increasing the extract concentration does not necessarily translate into greater barrier-protective activity and highlights the importance of concentration optimization when evaluating the biological effects of polyphenol-rich extracts.

**Figure 4:**
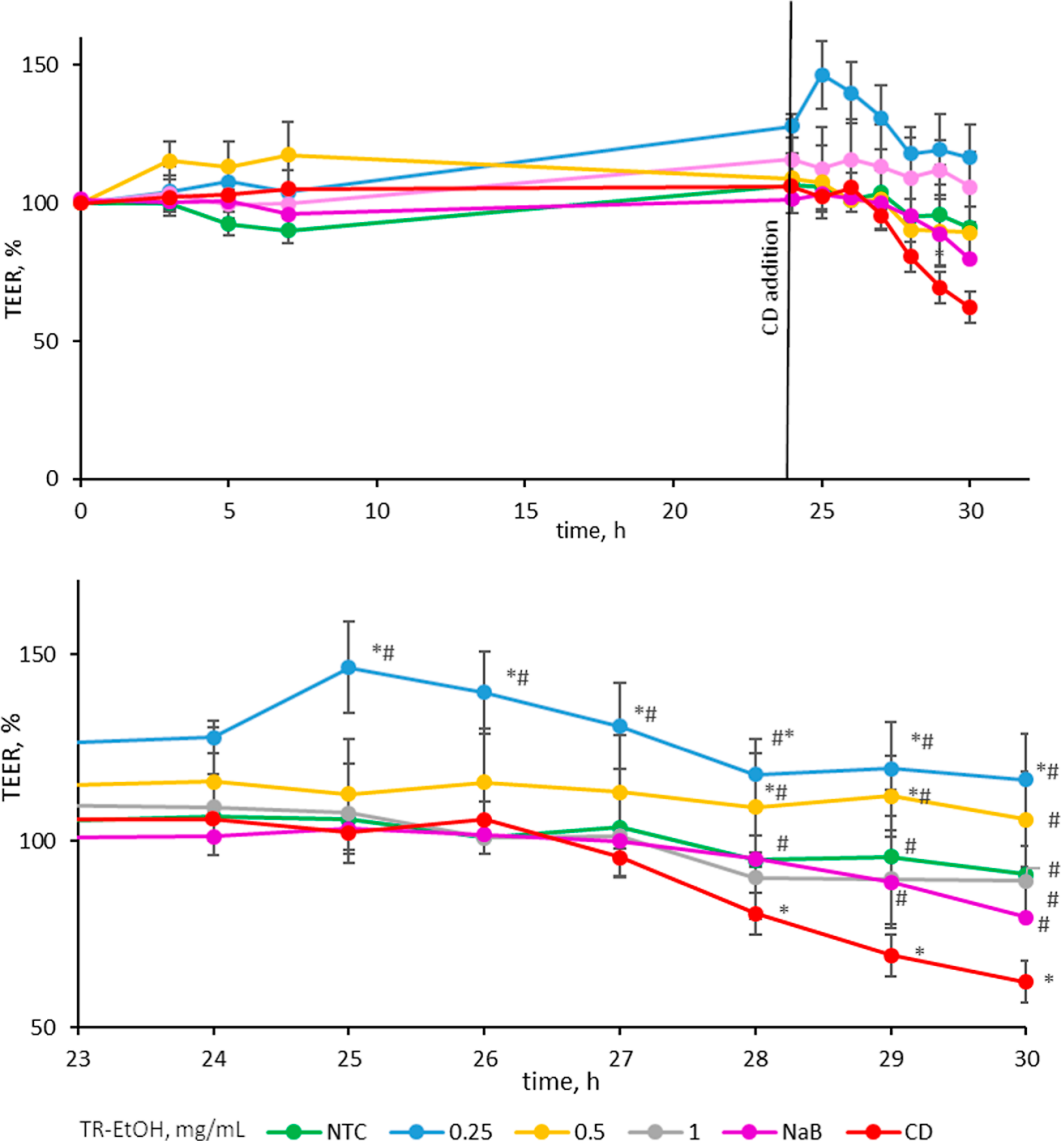
Effect of TR-EtOH (0.25–1 mg/mL) on TEER value of Caco-2 monolayers before and after stimulation with the TcdA/TcdB mixture (2.5 and 0.8 ng/mL, respectively). Toxins were added at 24 h, while TREMs samples were applied at time 0 h. Lower graph present a zoomed-in view of the 22–30 h time frame. TEER values are expressed as % of baseline. As a positive control sodium butyrate (NaB, 5 mM) was used. Results are presented as mean ± SD (n = 3). Statistical significance was calculated using Dunnett’s post hoc test at *p* ≤ 0.05 versus nontreated control (NTC, *) or toxin-stimulated control (CD, #).

To evaluate how gut microbiota metabolism modifies the barrier-stabilizing properties of TR-EtOH, the effect of TREMs (equivalent to 1 mg/mL of extract) on TEER was tested under the same conditions as the parent extract. As presented in the upper part of Figure 5, similarly as for TR-EtOH, TEER remained stable during the 24 h preincubation with TREMs, indicating that none of them compromised epithelial barrier integrity. The slight initial decrease observed for some samples is likely related to medium exchange rather than a biological effect of the metabolites. After toxin addition (at 24 h), significant differences in protective activity were observed among TREMs (zoomed-in view in lower part of Figure 5). After 6 h of toxins exposure, TREM-D3-30 was the most effective fraction, maintaining the TEER value (90.4 ± 4.0%) close to that of the NTC (95.1 ± 3.6%), and significantly higher than the toxins-treated control (69.4 ± 5.6%). Its activity was comparable to NaB (88.9 ± 9.2%). At this time point, TREM-D2-30 and TREM-D2-100 exhibited moderate protective effects (78.5 ± 10.5 and 79.1 ± 3.5%, respectively), whereas the remaining fraction, TREM-D1-30, TREM-D1-100, and TREM-D3-100 (65.9 ± 4.7, 65.2 ± 3.1 and 67.3 ± 3.0%, respectively), did not show barrier-protective activity, as their TEER values were close to CD (69.4 ± 5.6%).

**Figure 5:**
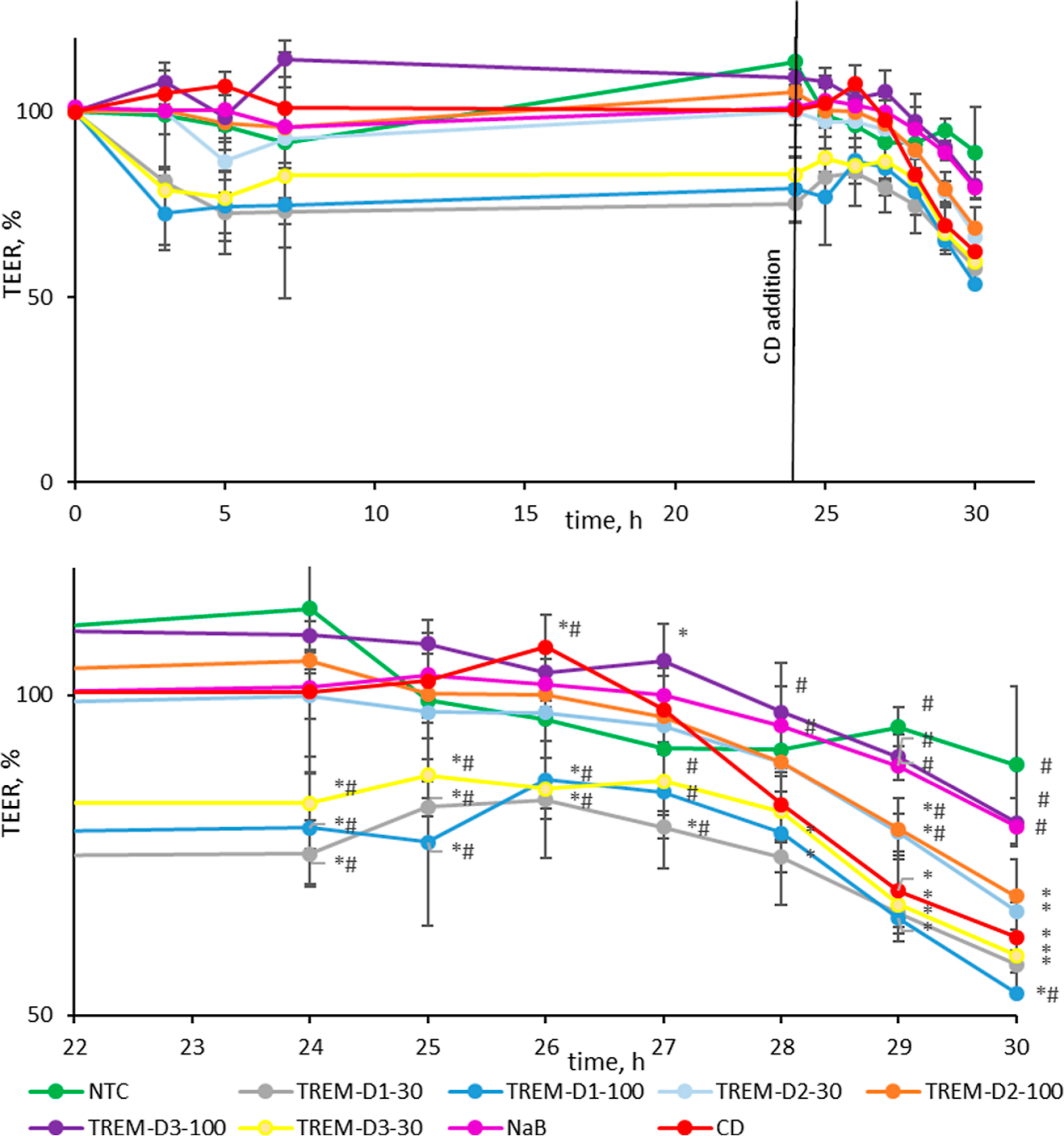
Effect of TREMs (equivalent to 1 mg/mL extract) on TEER value of Caco-2 monolayers before and after stimulation with the TcdA/TcdB mixture (2.5 and 0.8 ng/mL, respectively). Toxins were added at 24 h, while TREMs samples were applied at time 0 h. Lower graph present a zoomed-in view of the 22–30 h time frame. TEER values are expressed as % of baseline. As a positive control sodium butyrate (NaB, 5 mM) was used. Results are presented as mean ± SD (n = 3). Statistical significance was calculated using Dunnett’s post hoc test at *p* ≤ 0.05 versus nontreated control (NTC, *) or toxins-stimulated control (CD, #).

The differential activity observed among TREMs indicates that gut microbiota transformation of TR-EtOH constituents does not consistently preserve or enhance biological activity. On the contrary, only selected metabolites exhibited barrier-protective effects, supporting the concept that the gut microbiota metabolism acts as a selective modulator of polyphenol bioactivity. This aligns with previous findings for other tannin-rich plant extracts, where biotransformation can lead to either activation or inactivation of functional properties.^20,47,48^ Chemical characterization of the TREMs revealed a significant inverse correlation between TPC and IC_50_ values, further highlighting the importance of polyphenols in mediating barrier protection. Nevertheless, not all fractions with relatively high TPC showed strong activity, suggesting that not only the quantity but also the specific structural features and molecular identities of the phenolic metabolites are critical for efficacy. Moreover, TEER stabilization may not only reflect direct effects on TJ, but also indirect mechanisms such as antioxidant or anti-inflammatory actions, which help preserve epithelial integrity under stress conditions.^49,50^ The nonlinear dose–response relationship observed for TR-EtOH further supports the notion that higher concentrations do not necessarily translate into greater biological activity, with the strongest barrier-protective effects observed at 0.25–0.5 mg/mL. To better elucidate the molecular basis of the protective effects, additional mechanistic studies were conducted. Gene expression analysis (qPCR) and protein-level assessment (Western blot) of TJ components were performed to determine whether TR-EtOH and its microbial metabolites modulate the structural elements responsible for epithelial barrier integrity.

#### 3.4.2 Expression of Tight Junction Protein Genes

To investigate whether TR-EtOH and TREMs modulate the expression of genes involved in TJ regulation, quantitative PCR was performed on Caco-2 monolayers exposed to *C. difficile* toxins. Following 24 h preincubation with the test compounds, mRNA levels of four key TJ markers, claudin-2, claudin-4, occludin, and zonula occludens-1, were quantified. Expression values were normalized to the housekeeping gene β-actin and are presented as fold changes relative to nontreated control cells (NTC) (Figure 6).

**Figure 6:**
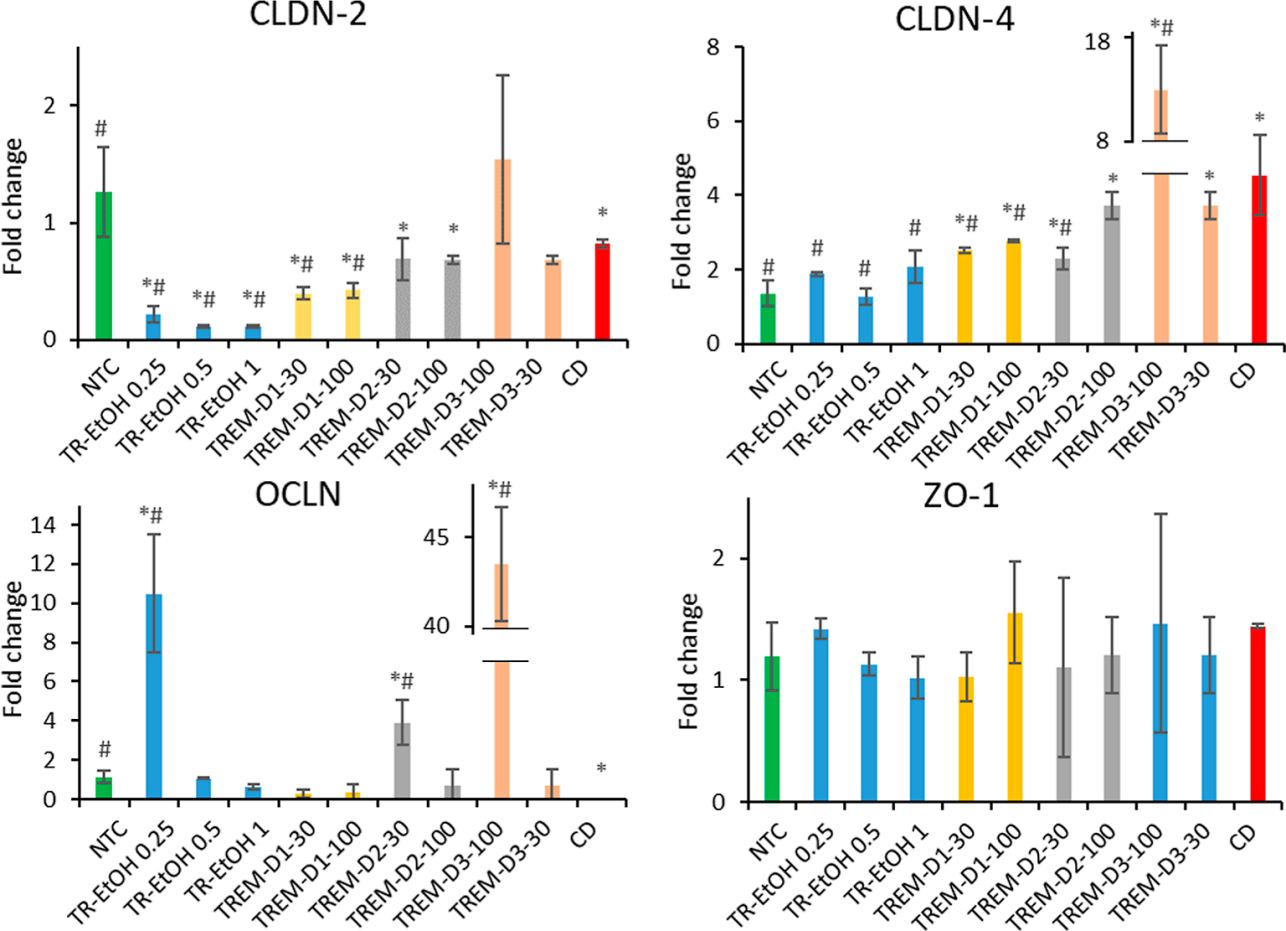
Effect of TR-EtOH (0.25–1 mg/mL) and TREMs (equivalent to 1 mg/mL extract) on the expression of tight junction protein genes: claudin-2 (CLDN-2), claudin-4 (CLDN-4), occludin (OCLN) and zonula occludens-1 (ZO-1), in Caco-2 cells treated with the TcdA/TcdB mixture (2.5 and 0.8 ng/mL, respectively). Gene expression levels were assessed by qPCR and normalized to β-actin. Data are expressed as fold change relative to the nontreated control (NTC). The inset shows TREM-D3-30 data at an expanded *y*-axis scale for clarity. Results are presented as mean ± SD (n = 3). Statistical significance was determined by Dunnett’s post hoc test at *p* ≤ 0.05 versus NTC (*) or the toxin-stimulated control (CD) (#).

Following exposure to *C. difficile* toxins, claudin-2, a pore-forming claudin associated with increased paracellular permeability, was significantly decreased (0.82 ± 0.03) compared to the nontreated control (1.26 ± 0.38). Pretreatment with TR-EtOH at concentrations 0.25, 0.5, and 1 mg/mL markedly suppressed CLDN-2 gene expression (0.22 ± 0.07, 0.12 ± 0.01, and 0.11 ± 0.02, respectively) in relation to CD and NTC. Among the gut microbiota metabolites, TREM-D1-30 and TREM-D1-100 significantly reduced CLDN-2 levels (0.40 ± 0.05 and 0.42 ± 0.07, respectively) compared to the toxins-treated and nontreated control. The remaining TREMs did not differ significantly from CD but TREM-D2-30 and TREM-D2-100 were significantly lower than NTC.

Exposure to *C. difficile* toxins increased expression of the claudin-4 gene, a barrier-sealing claudin in relation to nontreated control. TR-EtOH at concentrations 0.25, 0.5, and 1 mg/mL reduced CLDN-4 gene expression (1.87 ± 0.04, 1.26 ± 0.21, and 2.06 ± 0.44, respectively) compared to the toxins-treated control (4.53 ± 1.07) and restored it to levels close to the NTC (1.34 ± 0.35). Among the metabolite fractions, TREM-D1-30, TREM-D1-100, and TREM-D2-30 showed a similar reduction (2.51 ± 0.07, 2.78 ± 0.04, and 2.31 ± 0.29, respectively) compared to CD, with no significant differences from the NTC. TREM-D2-100 and TREM-D3-100 did not significantly differ from the toxins-treated control but were still significantly higher (3.71 ± 0.36 and 3.72 ± 0.36, respectively) than NTC. Notably, TREM-D3-30 resulted in a markedly elevated expression of CLDN-4 gene (12.88 ± 4.57), greatly exceeding all other groups.

For occludin, a core structural component of tight junctions, gene expression was significantly reduced after toxins exposure. TR-EtOH at 0.25 mg/mL strongly enhanced expression (10.48 ± 3.00), showing a significant increase compared to both the NTC (1.14 ± 0.29) and toxins-treated control (0.01 ± 0.01). Other concentrations of TR-EtOH, as well as TREM-D1-30, TREM-D1-100, TREM-D2-100, and TREM-D3-100, did not significantly differ from either control group. In contrast, TREM-D2-30 and TREM-D3-30 significantly increased OCLN gene expression (3.89 ± 1.13 and 43.62 ± 3.20, respectively) compared to both the NTC and CD, with TREM-D3-30 reaching particularly high expression levels.

Expression of zonula occludens-1, a cytoplasmic scaffolding protein critical for TJ assembly, remained largely unchanged across treatment groups, with high variability and no statistically significant differences between controls (1.20 ± 0.28 1.44 ± 0.01 for NTC and CD, respectively).

These findings indicate that the protective effects of TR-EtOH and selected TREMs may, at least in part, involve transcriptional regulation of TJ genes. The observed gene expression profile suggested modulation of genes involved in epithelial barrier regulation. Reduction of CLDN-2 expression, a claudin associated with increased paracellular permeability, was consistently observed with all TR-EtOH concentrations as well as TREM-D1-30 and TREM-D1-100. In contrast, TREM-D2-30 and TREM-D2-100 reduced CLDN-2 levels relative to the nontreated control only, without significant differences compared to the toxin-treated control. This pattern is in line with previous evidence showing that polyphenols can downregulate permeability-associated claudins.^51,52^ In contrast, CLDN-4, a barrier-sealing claudin, was restored to baseline by TR-EtOH and TREM-D1-30–TREM-D2-30, while TREM-D3-30 induced exceptionally high levels. The biological significance of these marked transcriptional responses remains unclear and warrants further investigation.^53,54^ Similarly, OCLN expression was strongly upregulated by low-dose TR-EtOH, TREM-D2-30, and especially TREM-D3-30. However, the biological significance of the exceptionally high response observed for TREM-D3-30 remains unclear, particularly given the lack of a corresponding increase at the protein level. In contrast, ZO-1 expression remained unchanged across experimental groups, showing high variability and no significant modulation at the transcriptional level. Similar observations have been reported previously for ZO-1 in epithelial barrier models.^55^ Taken together, the transcriptional profile observed in this study suggests that TR-EtOH and selected TREMs modulate the expression of genes involved in tight junction regulation and may contribute to protection against toxin-induced barrier disruption.

#### 3.4.3 Expression of Tight Junction Proteins

To assess the effect of TR-EtOH and TREMs on TJ disruption, the expression of key tight junction proteins in Caco-2 monolayers treated with *C. difficile* toxins was analyzed by Western blot method. Protein levels of claudin-2, claudin-4, occludin and zonula occludens-1 were determined after 24 h preincubation with test samples and normalized to β-actin. Results were expressed as fold change relative to the nontreated control (Figure 7). ZO-1 was not detectable in the gels, and thus the results are not shown. Representative Western blot images are provided in Supporting Information, Figure S6.

**Figure 7:**
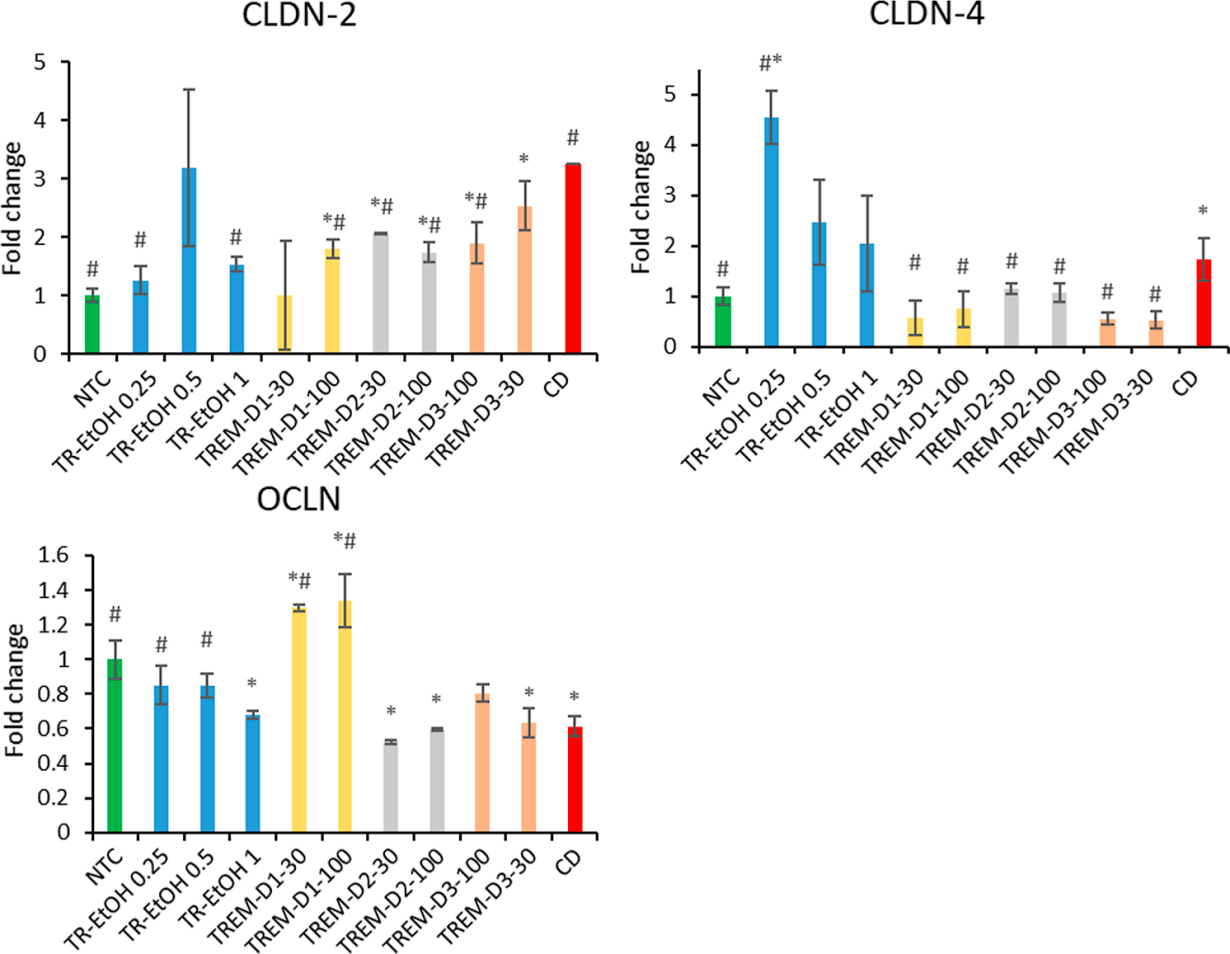
Effect of TR-EtOH (0.25–1 mg/mL) and TREMs (equivalent to 1 mg/mL extract) on the expression of tight junction proteins: claudin-2 (CLDN-2), claudin-4 (CLDN-4), and occludin (OCLN), in Caco-2 cells stimulated with the TcdA/TcdB mixture (2.5 and 0.8 ng/mL, respectively). Protein expression levels were assessed by Western blot and normalized to β-actin. Data are expressed as fold change relative to the nontreated control (NTC). Results are presented as mean ± SD (n = 3). Statistical significance was determined by Dunnett’s post hoc test at *p* ≤ 0.05 versus NTC (*) or the toxin-stimulated control (CD) (#).

*C. difficile* toxins significantly increased the expression of CLDN-2 (3.25 ± 0.01), compared to the nontreated control (1 ± 0.12). Treatment with TR-EtOH (0.25 and 1 mg/mL) significantly reduced CLDN-2 expression following toxins exposure (1.26 ± 0.23 and 1.53 ± 0.14, respectively). Similar effect was observed for TREM-D1-100, TREM-D2-30, TREM-D2-100 and TREM-D3-30 which decreased CLDN-2 production (1.80 ± 0.17, 2.05 ± 0.01, 1.74 ± 0.18, and 1.89 ± 0.35, respectively), compared to the nontreated and toxins-treated controls. TR-EtOH at concentration 0.5 mg/mL, TREM-D1-30 and TREM-D3-100 did not differ significantly from CD, but TREM-D3-100 was significantly decreased compared to NTC.

Production of CLDN-4 was also elevated following toxins exposure (1.72 ± 0.43), compared to nontreated control (1.00 ± 0.18). TR-EtOH, at 0.25 mg/mL, significantly enhanced CLDN-4 expression (4.54 ± 0.53), in comparison to NTC and CD. Remaining extract concentrations did not differ significantly from nontreated and toxins-treated control. In contrast, all TREMs significantly reduced CLDN-4 levels (0.58 ± 0.34, 0.74 ± 0.37, 1.16 ± 0.11, 1.07 ± 0.18, 0.55 ± 0.11, and 0.52 ± 0.17, for TREM-D1-30-6, respectively), relative to toxins-treated control and did not differ from nontreated control.

Addition of toxins from *C. difficile* significantly reduced occludin expression (0.61 ± 0.06) compared to nontreated control (1.00 ± 0.11). TR-EtOH at concentrations 0.25 and 0.5 mg/mL significantly increased OCLN production in comparison to CD to level close to NTC. Among TREMs, only TREM-D1-30 and TREM-D1-100 led to enhanced expression of occludin (0.85 ± 0.11, and 0.85 ± 0.07, respectively) relative to NTC and CD. Remaining TREMs and TR-EtOH at concentration 1 mg/mL did not differ significantly from toxins-treated control but were significantly lower than nontreated control (except TREM-D3-30).

The Western blot results complement TEER and gene expression qPCR analyses, providing mechanistic insight into how TR-EtOH and its gut microbiota-derived metabolites protect intestinal barrier integrity. *C. difficile* toxins induced a typical pattern of TJ disruption: CLDN-2 upregulation, OCLN downregulation, and compensatory induction of CLDN-4, in line with previous reports describing toxins-mediated barrier destabilization.^56^ Pretreatment with TR-EtOH effectively counteracted these changes by downregulating CLDN-2, restoring OCLN, and markedly enhancing CLDN-4, consistent with the transcriptional profile and TEER stabilization. These effects are likely linked to its high polyphenol content, particularly procyanidins and ellagic acid derivatives, which have been shown to modulate NF-κB and MAPK signaling and thereby maintain epithelial integrity under oxidative and inflammatory stress.^51,52^ In contrast, the activity of TREMs displayed greater variability, reflecting donor-dependent metabolic patterns. While almost all metabolite fractions reduced CLDN-2 protein levels, their effects on CLDN-4 and OCLN were generally restorative rather than stimulatory. Most TREMs brought protein expression back to baseline levels observed in untreated cells, rather than exceeding them as seen with TR-EtOH. This indicates that gut microbiota metabolism may attenuate the direct barrier-sealing properties of the parent extract but preserve its ability to mitigate increases in permeability. Interestingly, fractions such as TREM-D2-30 and TREM-D3-30 showed stronger and more consistent effects, which is in line with their relatively higher TPC and antioxidant activity. This suggests that not only the total phenolic content, but also the structural nature of metabolites (e.g., catechin derivatives, small phenolic acids) may contribute to the observed differences in bioactivity.^57^ The results obtained at the protein level generally confirmed the transcriptional trends observed in qPCR, yet also revealed notable discrepancies that highlight the complexity of TJ regulation. For CLDN-2, partial agreement between gene and protein analyses was observed. TR-EtOH showed consistent effects across both levels, whereas the correspondence for individual TREM fractions was less pronounced. In contrast, the regulation of CLDN-4 showed divergence. While qPCR analysis revealed that TR-EtOH and TREM-D1-30-3 restored its expression to baseline and TREM-D3-30 induced an exceptionally strong transcriptional overshoot, the protein analysis demonstrated that only TR-EtOH (0.25 mg/mL) enhanced CLDN-4, whereas all TREMs reduced its level close to control values. Similarly, for occludin, low-dose TR-EtOH and TREM-D2-30 and TREM-D3-30 markedly increased mRNA expression, but this effect was not reproduced at the protein level. Instead, protein restoration was observed with TR-EtOH (0.25 and 0.5 mg/mL) and TREM-D1-30 and TREM-D1-100. Notably, the recovery of occludin protein by TREM-D1-30 and TREM-D1-100 occurred without significant induction of OCLN mRNA, further illustrating the limited correspondence between transcript and protein abundance under these experimental conditions. Finally, ZO-1 remained unchanged at the gene level and was undetectable by Western blot analysis under the applied experimental conditions. Therefore, no conclusions regarding ZO-1 protein regulation can be drawn from the present data, and both biological and technical explanations for the lack of signal should be considered. Such discrepancies between transcriptional and translational data are not uncommon. Previous studies have shown that TJ regulation often depends more on protein localization, turnover, and phosphorylation than on absolute transcriptional changes.^58^ In polyphenol research, discrepancies between mRNA and protein levels have also been reported for flavonoids and tannins, highlighting the complexity of tight junction regulation.^18,59^ In this context, the strong induction of OCLN and CLDN-4 transcripts by TREM-D3-30 was not accompanied by a corresponding increase in protein expression. Conversely, the ability of TREM-D1-30 and TREM-D1-100 to restore occludin protein without significant transcriptional induction further supports the conclusion that mRNA and protein responses were not always directly coupled in the present model.^18,59^

Taken together, these findings highlight a complementary mode of action: TR-EtOH exerted stronger effects on TJ-related markers, whereas TREMs acted more moderately, buffering the disruptive effects of toxins and supporting epithelial homeostasis. Integration of antioxidant data, TEER, qPCR, and protein analyses underscores the importance of both dietary polyphenols and their gut microbiota-derived metabolites in protecting intestinal barrier function, although through partially distinct mechanisms.

### 3.5 Effects of TR-EtOH and TREMs on IL-8 Secretion in TcdA and TcdB-Stimulated Caco-2

To investigate the inflammatory response of intestinal epithelial cells to *C. difficile* toxins, Caco-2 cells were preincubated with TR-EtOH or TREMs for 24 h, followed by 6 h exposure to the toxins. The secretion of a panel of pro- and anti-inflammatory cytokines was then assessed. Although the full cytokine panel (including IL-12p70, TNF-α, IL-10, IL-6, and IL-1β) was assessed, these cytokines were detected only at very low or inconsistent levels, close to the assay’s detection limits. Therefore, only the interleukin 8 (IL-8) results are presented in the main text (Figure 8), while the remaining data are provided in Supporting Information (Figure S7). Stimulation with *C. difficile* toxins significantly increased IL-8 secretion (139.6 ± 17.2%) compared to the nontreated control (100.1 ± 4.5%). TR-EtOH at all tested concentrations (0.25–1 mg/mL) and all TREMs significantly reduced IL-8 secretion relative to the toxins-treated control. For TR-EtOH, this reduction was concentration-dependent (90.1 ± 13.4%, 80.4 ± 5.0%, and 72.6 ± 6.2% for 0.25, 0.5, and 1 mg/mL, respectively), whereas TREMs exhibited comparable inhibitory activity, with the strongest effect observed for TREM-D1-30, TREM-D2-30, TREM-D2-100, and TREM-D3-30 (68.8 ± 4.7, 62.7 ± 14.9%, 70.7 ± 5.5%, and 70.3 ± 5.5%, respectively), which were even lower than the nontreated control. Notably, for particular donor, more polar fractions eluted with 30% methanol demonstrated greater inhibitory activity compared to less polar fractions eluted with 100% methanol. Dexamethasone (20 μM), widely recognized for its anti-inflammatory properties, including stimulated Caco-2 cell models, was used as a positive control and effectively suppressed IL-8 secretion, confirming assay sensitivity.^30^

**Figure 8:**
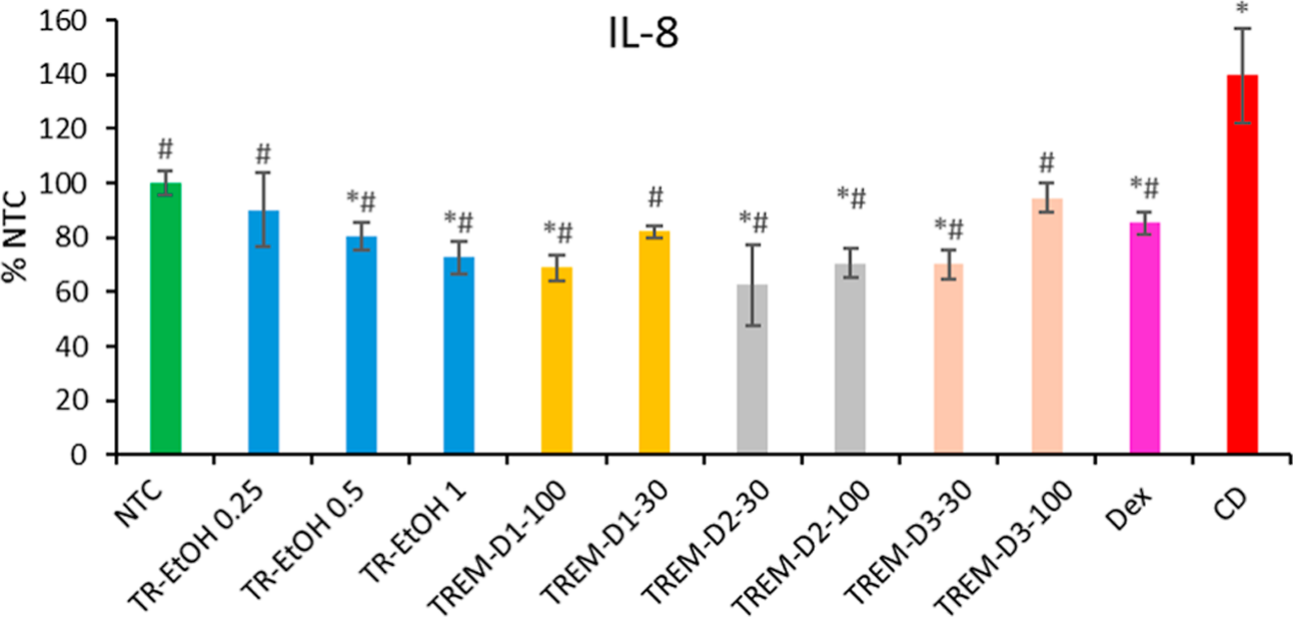
Effect of TR-EtOH (0.25–1 mg/mL) and TREMs (equivalent to 1 mg/mL extract) on IL-8 production in Caco-2 cells stimulated with the TcdA/TcdB mixture (2.5 and 0.8 ng/mL, respectively). IL-8 levels are expressed as a percentage relative to the nontreated control (NTC). As a positive control dexamethasone (Dex, 20 μM) was used. Data are presented as mean ± SD (n = 3). Statistical significance was determined by Dunnett’s post hoc test at *p* ≤ 0.05 versus NTC (*) or the toxin-stimulated control (CD) (#).

Interleukin 8 is a key chemokine involved in neutrophil recruitment and a reliable marker of epithelial inflammation.^60^ Its significant upregulation following exposure to *C. difficile* toxins supports its utility as a primary marker of inflammation in the Caco-2 model. The low or inconsistent detection of other cytokines (e.g., IL-6, TNF-α, IL-10) is consistent with the limited immune response of Caco-2 cells reported in the literature.^61,62^ Therefore, the inhibitory effects observed in this study should be interpreted primarily in the context of epithelial IL-8 regulation rather than as a comprehensive assessment of immune modulation. In the present study, both TR-EtOH and TREMs significantly attenuated IL-8 secretion in Caco-2 cells stimulated with *C. difficile* toxins, in line with expectations based on previously published data characterizing their phytochemical composition and the known anti-inflammatory potential of their major constituents.^24^ Among these, procyanidins are likely the main contributors to the extract’s anti-inflammatory effects, while triterpenes and ellagic acid derivatives may play a complementary role through mechanisms such as COX inhibition or redox modulation.^63–65^ Notably, more polar TREMs fractions eluted with 30% methanol showed greater inhibitory activity than less polar 100% methanol fractions. This corresponds to their composition, as the 30% MeOH fractions contained catechin oligomers and their gut microbiota-derived metabolites, which are polar compounds known for anti-inflammatory activity and good availability to epithelial cells. In contrast, the 100% MeOH fractions were dominated by triterpenes, lower-mass catechin metabolites, and small amount of unmetabolized ellagic acid derivatives.^24^ Although these compounds may contribute to the observed effects, their lower polarity could limit uptake by epithelial cells, and their activity might involve mechanisms not directly linked to IL-8 secretion.^66,67^ Notably, the inhibitory effect for IL-8 of TR-EtOH and its polar TREMs fractions were consistent with their antioxidant potential and polyphenol content. Samples showing the strongest inhibition of IL-8 secretion, such as TR-EtOH, TREM-D1-30, TREM-D2-30 and TREM-D3-30, also exhibited the highest TPC and lowest IC_50_ values in the DPPH assay. This correlation supports a possible association between phenolic content, antioxidant capacity and attenuation of IL-8 secretion, however, the underlying mechanisms remain to be established.

### 3.6 Limitations

A limitation of the present study is the use of *Clostridioides difficile* toxins as a model of epithelial barrier dysfunction. Although TcdA and TcdB provide a rapid and reproducible method for inducing tight junction disruption, they act through a specific mechanism involving Rho GTPase inactivation and cytoskeletal remodeling. Consequently, this model does not fully reflect the multifactorial nature of intestinal barrier dysfunction associated with chronic inflammatory and metabolic disorders. Nevertheless, impairment of tight junction integrity is a common feature of increased intestinal permeability irrespective of the initiating stimulus, making this model suitable for investigating barrier-protective effects of dietary polyphenols and their gut microbiota-derived metabolites. Future studies employing complementary inflammatory models and in vivo systems are warranted to further assess the translational relevance of these findings. Another limitation of the present study is that dose–response relationships were evaluated only for TR-EtOH, whereas TREMs were tested at a single relative concentration corresponding to metabolites generated from the parent extract during fermentation. Therefore, the relative potency of individual metabolite fractions could not be assessed and should be addressed in future studies.

This study demonstrates that TR-EtOH and its gut microbiota-derived metabolites exert complementary protective effects on intestinal epithelial cells exposed to *C. difficile* toxins. Gut microbiota metabolism reduced the total phenolic content and antioxidant potential of TR-EtOH but generated metabolites that retained moderate and distinct bioactivity. Functionally, TR-EtOH strongly stabilized the epithelial barrier and suppressed inflammation, while TREMs showed more variable but still relevant barrier-protective and anti-inflammatory effects. These findings highlight the complementary contribution of the parent extract and its gut microbiota-derived metabolites to intestinal barrier protection and modulation of epithelial inflammatory responses. However, further studies in more physiologically relevant models, including in vivo systems, are required to confirm the translational relevance of these observations and their potential application in functional foods or beverages.

## Supplementary Material



## Notes

Primer sequences used for qPCR analysis; UHPLC-DAD-MS/MS data of compounds detected in the tormentil rhizome ethanolic extract and gut microbiota-derived metabolite fractions; representative TEER profiles of Caco-2 monolayers following exposure to deoxynivalenol, TNF-α, IFN-γ, lipopolysaccharide, and mefenamic acid; effects of TcdA/TcdB and dexamethasone on Caco-2 cell viability; representative Western blot images of Caco-2 cells; effects of TR-EtOH and TREMs on the production of IL-12p70, TNF-α, IL-10, IL-6, and IL-1β (PDF)

## Abbreviations

ACTB: β-actin
ANOVA: analysis of variance
BHI: brain heart infusion broth
CD: *Clostridioides difficile* toxins-treated control
CLDN-2: claudin-2
CLDN-4: claudin-4
DAD: diode array detector
Dex: dexamethasone
DMSO: dimethyl sulfoxide
DON: deoxynivalenol
DPPH: 2,2-diphenyl-1-picrylhydrazyl
ELISA: enzyme-linked immunosorbent assay
ESI: electrospray ionization
GA: gallic acid
HRP: horseradish peroxidase
IC_50_: half maximal inhibitory concentration
IFN-γ: interferon gamma
IL-8: interleukin 8
JAK-STAT: Janus kinase-signal transducer and activator of transcription
LA: l-ascorbic acid
LPS: lipopolysaccharide
MA: mefenamic acid
MAPK: mitogen-activated protein kinase
MEM: minimum essential medium
MS: mass spectrometry
MS/MS: tandem mass spectrometry
MTT: 3-(4,5-dimethylthiazol-2-yl)-2,5-diphenyltetrazolium bromide
NaB: sodium butyrate
NF-κB: nuclear factor kappa B
NTC: nontreated control
OCLN: occludin
PBS: phosphate-buffered saline
qPCR: quantitative polymerase chain reaction
SDS-PAGE: sodium dodecyl sulfate–polyacrylamide gel electrophoresis
TBS: Tris-buffered saline
TBP: TATA-box binding protein
TcdA: toxin A (*Clostridioides difficile* toxin A)
TcdB: toxin B (*Clostridioides difficile* toxin B)
TEER: transepithelial electrical resistance
TJP: tight junction protein
TJ: tight junction
TNF-α: tumor necrosis factor alpha
TPC: total phenolic content
TR-EtOH: tormentil rhizome ethanolic extract
TREMs: tormentil rhizome ethanolic extract metabolites
UHPLC: ultrahigh-performance liquid chromatography
UV: ultraviolet
ZO-1: zonula occludens-1.
