# Supplementary material for: Tormentil Rhizome Ethanolic Extract and Its Gut Microbiota-Derived Metabolites: Modulation of Tight Junction Integrity and Anti-Inflammatory Potential in Caco‑2 Cells
Source: J Agric Food Chem. 2026 Jul 19;74(29):22859–76. doi: 10.1021/acs.jafc.6c03087 (PMC13426303; doi:10.1021/acs.jafc.6c03087)
Supplement: Supplementary file 1 [file jf6c03087_si_001.pdf]

Supporting Information

Tormentil Rhizome Ethanolic Extract and Its Gut Microbiota-Derived Metabolites: Modulation of Tight Junction Integrity and Anti-Inflammatory Potential in Caco-2 Cells

Aleksandra Kruk[a]*, Łukasz Grześkowiak[b], Inna Vlasova[c], Yuliia Kostenko[c], Jürgen Zentek[b], Sebastian Granica[a], Jakub P. Piwowarski[c]

[a]Department of Pharmaceutical Biology, Faculty of Pharmacy, Medical University of Warsaw, Banacha 1b Street, Warsaw 02-097, Poland

[b]Institute of Animal Nutrition, Freie Universitat Berlin, Konigin-Luise 49 Street, Warsaw 14195, Germany

[c]Department of Pharmaceutical Biology and Bioanalysis, Faculty of Pharmacy, Medical University of Warsaw, Banacha 1b Street, Warsaw 02-097, Poland

*aleksandra.kruk@wum.edu.pl

Table S1. Sequences of primers used in the study, along with their annealing temperatures and expected product lengths.

PCR-Produkt	Gen	Annealing temp.	length (bp)	Primer 1	Sequence (5' - 3')	Primer 2	Sequence (5' - 3')
ACTB	β-Actin	60°C	129	ACT-h-f1	TTGCCGACAGGATGCAGAAGGA	ACT-h-r1	AGGTGGACAGCGAGGCCAGGAT
TBP	TATA boxbinding protein	60°C	132	TBP-h-f1	TGCACAGGAGCCAAGAGTGAA	TBP-h-r1	CACATCACAGCTCCCCACCA
OCLN	Ocludin	60°C	105	OCLDNh-f1	TCCTATAAATCCACGCCGGTTC	OCLDNh-r1	CTCAAAGTTACCACCGCTGCTG
ZO-1	Zona ocludens 1	60°C	184	ZO1h-f1	CGGGACTGTTGGTATTGGCTAGA	ZO1h-r1	GGCCAGGGCCATAGTAAAGTTTG
CLDN-4	Claudin 4	60°C	103	CLDN4h-f2	CATCGGCAGCAACATTGTCA	CLDNrh-f2	CGAGTCGTACACCTTGCACT
CLDN-2	Claudin 2	58°C	175	CLDN2h-f4	CCTGGGATTCATTCCTGTTG	CLDN2h-r4c	CTGGGATGAGCAGGAAAAGC

Table S2. UHPLC-DAD-MS/MS data of compounds detected in the raw extract and their quantitative analysis. a – $[M-2H]^{2-}$; b – base peak; c – $[M+HCOOH-H]^{-}$. The content was expressed as mg of compound per g of dry extract. LOD – Limit of Detection; LOQ – Limit of Quantification; n/a – not applicable. Presented data was based on previously published analyses:
Kruk A, Popowski D, Roszko M, Granica S, Piwowarski JP. Heterogeneity of transport and metabolism of Tormentillae rhizoma constituents across human intestinal epithelium cellular model. Food Res Int. 2024;188:114326. doi:10.1016/j.foodres.2024.114326
Kruk A, Popowski D, Średnicka P, Roszko MŁ, Granica S, Piwowarski JP. Selective metabolism of tormentil rhizome constituents by human gut microbiota and its impact on biodiversity ex vivo. Food Chem. 2025;478:143674. doi:10.1016/j.foodchem.2025.143674

t_r, min	UV max, nm	*m*/*z*	MS2	Reference Ion	Identification	Content, mg/g
5.5	275	315	123, 153b, 297	$[M-H]^{-}$	protocatechuic acid *O*-hexoside	<LOQ
10.6	278	865	287, 289, 451, 543, 475, 577, 695b, 713, 739	$[M-H]^{-}$	procyanidin trimer type C isomer I	<LOQ
10.9	279	451	161, 179, 245, 289b	$[M-H]^{-}$	catechin-*O*-hexoside	<LOQ
13.0	279	577	245, 287, 289, 407b, 425, 451,	$[M-H]^{-}$	procyanidin dimer type B isomer I	95.45
13.9	278	289	125, 160, 179, 203, 205, 245b	$[M-H]^{-}$	catechin	19.57
15.2	279	865	287, 289, 451, 543, 475, 577, 695b, 713, 739	$[M-H]^{-}$	procyanidin trimer type C isomer II	8.53
18.5	278	561	289b, 329, 407, 543	$[M-H]^{-}$	catechin and (epi)afzelechin dimer	<LOQ
20.7	279	576	245, 287, 289b, 407, 425, 451, 491, 559	$[M-2H]^{2-}$	procyanidin tetramer isomer I	7.61
21.9	279	577	245, 287, 289, 407b, 425, 451	$[M-H]^{-}$	procyanidin dimer type B isomer II	8.87
22.5	278	441	249, 395b	$[M + HCOOH-H]^{-}$	unknown	n/a
23.7	277	577	245, 287, 289, 407b, 425, 451	$[M-H]^{-}$	procyanidin dimer type B isomer III	<LOQ
23.7	277, 371	615	301b, 463	$[M-H]^{-}$	ellagic acid *O*-(*O*-galloyl)-hexoside	0.17

t_r, min	UV max, nm	*m*/*z*	MS2	Reference Ion	Identification	Content, mg/g
24.7	278	720	245, 287, 289, 407, 425, 451, 575, 577b, 636, 695	$[M-2H]^{2-}$	procyanidin pentamer isomer I	<LOQ
25.5	278	576	245, 287, 289b, 407, 425, 451, 491, 559	$[M-2H]^{2-}$	procyanidin tetramer isomer II	<LOQ
26.4	278	576	245, 287, 289b, 407, 425, 451, 491, 559	$[M-2H]^{2-}$	procyanidin tetramer isomer III	4.58
27.3	278, 364	463	301b	$[M-H]^{-}$	ellagic acid *O*-hexoside	0.51
27.3	278	577	245, 287, 289b, 407, 425, 451	$[M-H]^{-}$	procyanidin dimer type B isomer IV	<LOQ
27.5	278	576	245, 287, 289b, 407, 425, 451, 491, 559	$[M-2H]^{2-}$	procyanidin tetramer isomer IV	<LOQ
29.0	278	377	179, 331b	$[M-H]^{-}$	gallic acid derivative	<LOQ
30.4	278	720	245, 287, 289, 407, 425, 451, 575, 577b, 636, 695	$[M-2H]^{2-}$	procyanidin pentamer isomer II	<LOQ
30.9	278	511	465b	$[M + HCOOH-H]^{-}$	unknown	n/a
32.4	278	576	245, 287, 289, 407, 425, 451, 491b, 559	$[M-2H]^{2-}$	procyanidin tetramer isomer V	<LOQ
32.4	278	865	287, 289, 451, 543, 575, 577, 695b, 713, 739	$[M-H]^{-}$	procyanidin trimer type C isomer III	<LOQ
33.7	250, 362	433	301b	$[M-H]^{-}$	ellagic acid *O*-pentoside	0.96
33.7	250, 362	477	300, 315b	$[M-H]^{-}$	methylellagic acid *O*-hexoside	0.42

t_r, min	UV max, nm	*m*/*z*	MS2	Reference Ion	Identification	Content, mg/g
34.0	278	720	245, 287, 289, 407, 425, 451, 575, 577b, 636, 695	$[M-2H]^{2-}$	procyanidin pentamer isomer III	<LOQ
34.3	252, 365	491	300, 315b	$[M-H]^-$	methylellagic acid *O*-glucuronide	0.53
35.5	252, 365	301	–	$[M-H]^-$	ellagic acid	0.77
40.9	276, 370	934	–	$[M-2H]^{2-}$	agrimoniin	<LOQ
42.1	254, 364	447	300, 315b	$[M-H]^-$	methyellagic acid *O*-pentoside isomer I	1.13
43.9	254, 364	447	300, 315b	$[M-H]^-$	methyellagic acid *O*-pentoside isomer II	0.06
45.0	278, 371	575	229, 287, 394, 449b	$[M-H]^-$	unknown flavonoid derivative	2.02
45.8	277, 371	435	273b	$[M-H]^-$	phlorizin	<LOQ
50.2	277	711	441, 503b, 665	$[M-H]^-$	unknown	n/a
51.1	278	605	229, 289, 315b, 453	$[M-H]^-$	unknown	n/a
53.1	277	507	149, 293, 317, 339, 443, 461b	$[M + HCOOH-H]^-$	unknown	n/a
53.5	277	493	149, 191, 221, 251, 293, 311, 315, 447b	$[M + HCOOH-H]^-$	unknown	n/a
54.8	277	493	149, 161, 221, 251, 263, 293, 447b	$[M + HCOOH-H]^-$	unknown	n/a
56.5	277	507	163, 234, 265, 307, 325, 461b	$[M + HCOOH-H]^-$	unknown	n/a

t_r, min	UV max, nm	*m*/*z*	MS2	Reference Ion	Identification	Content, mg/g
66.4	219	695	469, 487b, 649	$[M + HCOOH-H]^-$	2,3,19-trihydroxy-urs-12-en-28-oic acid-28-*O*-D-glucopyranoside isomer (tormentic acid *O*-D-glucopyranoside isomer)	7.51
67.2	219	695	469, 487b, 533, 649	$[M{-}H]^-$	2,3,19,23-tetrahydroxy-11-methoxy-urs-12-en-28-oic acid-28-*O*-D-glucopyranoside	8.69
68.5	219	695	469, 487b, 649	$[M + HCOOH-H]^-$	2α,3β,19α-trihydroxy-urs-12-en-28-oic acid-28-*O*-β-D-glucopyranoside isomer II (tormentic acid *O*-β-D-glucopyranoside)	26.47
73.4	220	695	469, 487b, 649	$[M + HCOOH-H]^-$	trihydroxy-urs-12-en-28-oic acid-28-*O*-hexoside isomer	<LOQ
74.0	220	693	467, 485b, 647	$[M{-}H]^-$	2,19-dihydroxy-3-oxo-urs-12-en-3–28- oic acid-28-*O*-D-glucopyranoside	6.17
74.3	220	693	467, 485b, 647	$[M{-}H]^-$	dihydroxy-oxo-urs-12-en-28 oic acid-*O*-hexoside isomer	1.75
76.2	220	503	441b, 485	$[M{-}H]^-$	myrianthic acid	<LOD
77.4	221	679	471b, 633	$[M + HCOOH-H]^-$	undefined triterpenoic acid hexoside	0.52
79.8	221	517	439, 455b	$[M{-}H]^-$	cecropiacic acid	<LOD

t_r, min	UV max, nm	*m/z*	MS2	Reference Ion	Identification	Content, mg/g
82.2	220	487	407, 423, 469b	[M−H]$^-$	2,3,19-trihydroxy-urs-12-en-28-oic acid isomer I (tormentic acid isomer I)	7.69
82.6	221	487	407, 423, 469b	[M−H]$^-$	2,3,19-trihydroxy-urs-12-en-28-oic acid isomer II (tormentic acid isomer II)	0.96
87.2	222	487	371, 423, 469b	[M−H]$^-$	trihydroxy-urs-12-en-28-oic acid isomer	<LOD
88.7	222	485	369, 423, 467b	[M−H]$^-$	dihydroxy-oxo-urs-12-en-28 oic acid isomer	1.63

Table S3. UHPLC-MS/MS data of detected compounds; b – base peak; d – [M+Cl]$^-$ with the peaks area ratio; E – eksperymental sample, C – control sample, D1–3 - donor number. Metabolite identities and fragmentation patterns were originally reported in Kruk A, Popowski D, Średnicka P, Roszko MŁ, Granica S, Piwowarski JP. Selective metabolism of tormentil rhizome constituents by human gut microbiota and its impact on biodiversity ex vivo. Food Chem. 2025;478:143674. doi:10.1016/j.foodchem.2025.143674. Peak area ratios were calculated in the present study.

t_r, min	MS-/ [M-H]$^-$	MS2	Identification	Peak area ratio					
				30% MeOH, E,D1 / 30% MeOH, C,D1	100% MeOH, E,D1 / 100% MeOH, C,D1)	30% MeOH, E,D2 / 30% MeOH, C,D2	100% MeOH, E,D2 / 100% MeOH, C,D2	30% MeOH, E,D3 / 30% MeOH, C,D3	100% MeOH, E,D3 / 100% MeOH, C,D3
**11.45**	291.08488	146.94, 207.06, 231.93, 235.92b	catechin derivative	201.038	50.912	1270.128	1375.302	2749.644	5813.788
**11.46**	354.07982	146.94, 207.06 231.93, 235.93, 290.07, 291.08b, 292.09, 327.06	procyanidin type B or C derivative	41.469	12.012	223.052	405.749	1519.589	1113.032

**12.39**	369.07126d	146.84, 190.93, 231.93, 245.92, 289.07b, 315.08	procyanidin type B or C derivative	69.652	308.519	149.778	1367.575	18.646	56.427
**12.39**	396.08992	146.93, 231.93, 235.92, 289.07b, 316.08, 333.09, 369.07	catechin derivative	54.201	409.398	128.305	1421.337	23.692	92.015
**12.75**	396.09004	146.94, 231.93, 235.92, 289.67, 369,07b	procyanidin type B or C derivative	7.249	84.05	17.411	298.278	1.756	18.265
**13.23**	167.03356	112.98, 132.87, 145.93, 146.94b	catechin derivative	18.321	120.181	14.489	149.426	91.112	80.145
**14.71**	371.08692d	146.84, 207.93, 231.93, 292.09, 291.08b, 336.11	catechin derivative	1.016	10.793	3.112	216.26	28.917	858.018
**14.71**	398.10561	146.94, 190.93, 231.93, 235.92, 291.08b, 317.10, 335.11, 371.09	procyanidin type B or C derivative	1.016	11.344	2.842	202.84	24.638	837.464
**14.74**	291.08489	146.93, 190.93, 231.93, 235.92b	catechin derivative	1.067	16.885	13.501	432.436	123.957	1521.376
**15.21**	315.08467	146.94, 190.92, 231.93, 236.92, 245.05b, 300.99	catechin derivative	2.205	68.76	4.651	134.693	2.05	15.974
**19.64**	271.05885	116.92, 146.94, 174.95, 190.93, 235.92b	catechin derivative	0.978	104.33	0.936	107.622	0.147	5.508
**19.80**	273.07454	112.98, 116.92, 146.94, 235.92b	catechin derivative	0.938	285.36	0.983	263.573	0.981	182.091
**20.02**	285.03821	112.98, 132, 87, 146.94, 190.93, 215.13b	catechin derivative	0.979	21.942	0.929	27.991	0.506	0.117
**20.90**	209.08021	112.98, 160.93, 132.87, 149.93, 160.85, 162.84b	catechin derivative	0.92	77.788	1.011	31.026	1.054	182.794
**22.06**	255.06413	160.84, 162.84, 197.81, 219.84b, 221.40	catechin derivative	1.134	85.507	0.97	59.85	0.623	84.479

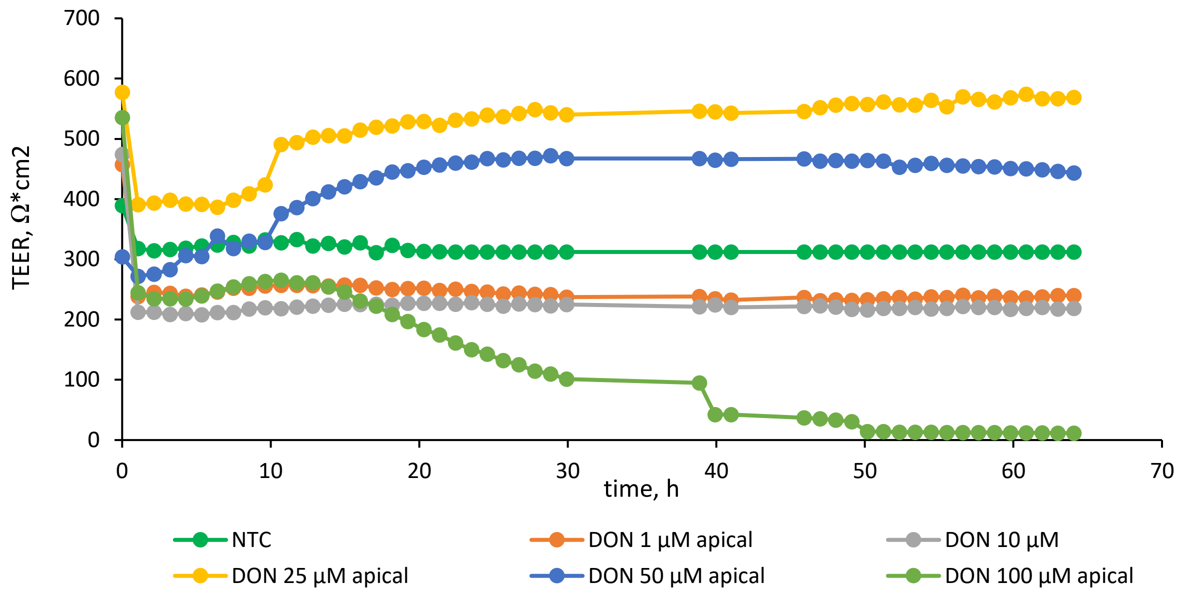

Figure S1. Representative TEER profiles of Caco-2 monolayers after exposure to the mycotoxin deoxynivalenol (DON), added at 0 h at concentrations of 1, 10, 25, 50 and 100 µM to apical side. NTC – non-treated control. For clarity, representative curves from three independent experiments are shown.

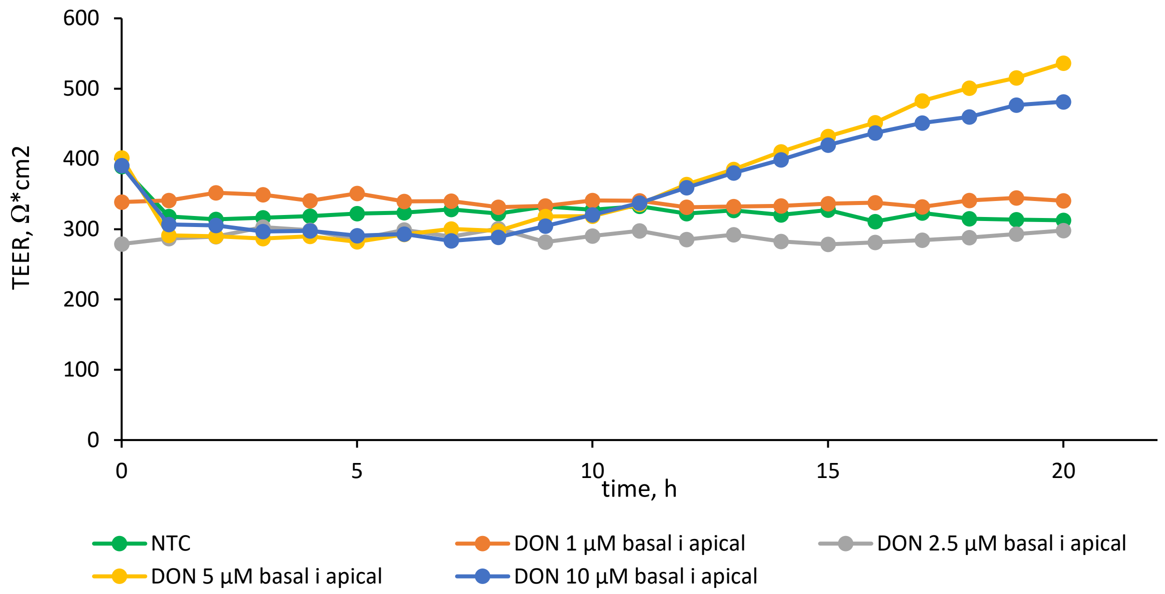

Figure S2. Representative TEER profiles of Caco-2 monolayers after exposure to the mycotoxin deoxynivalenol (DON), added at 0 h at concentrations of 1, 2.5, 5, and 10 µM to apical and basal side. NTC – non-treated control. For clarity, representative curves from three independent experiments are shown.

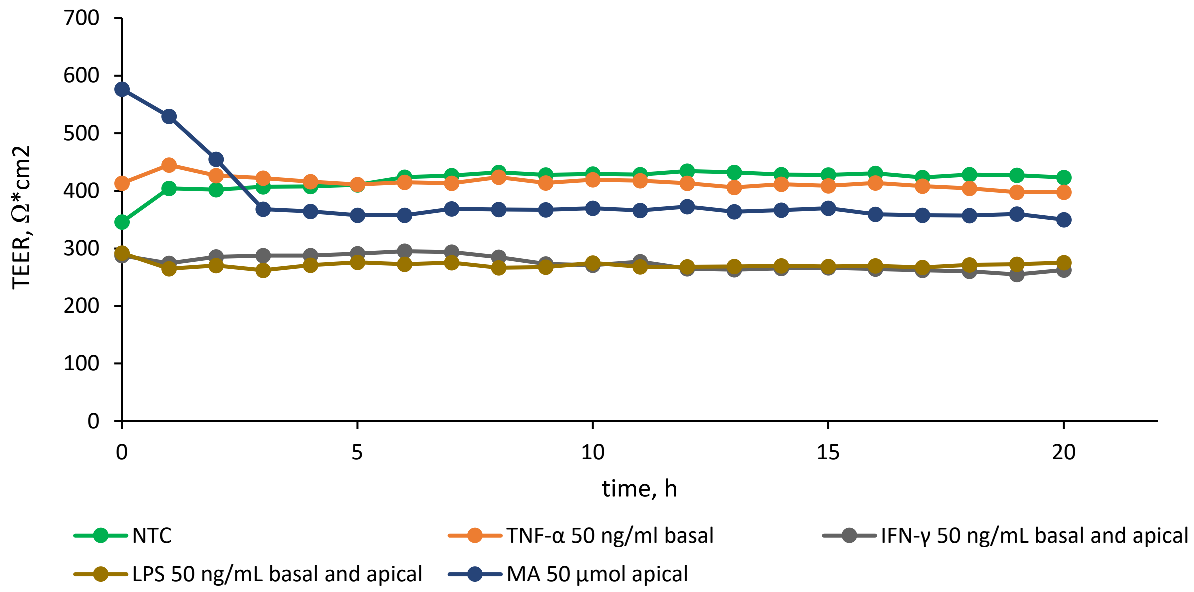

Figure S3. Representative TEER profiles of Caco-2 monolayers after exposure to various tight junction-disrupting factors, added at 0 h. Tested stimuli included pro-inflammatory cytokines (e.g. TNF-α, IFN-γ), bacterial components (LPS), and the non-steroidal anti-inflammatory drug mefenamic acid (MA) at different concentrations and polarities (basal/apical). NTC – non-treated control. For clarity, representative curves from three independent experiments are shown.

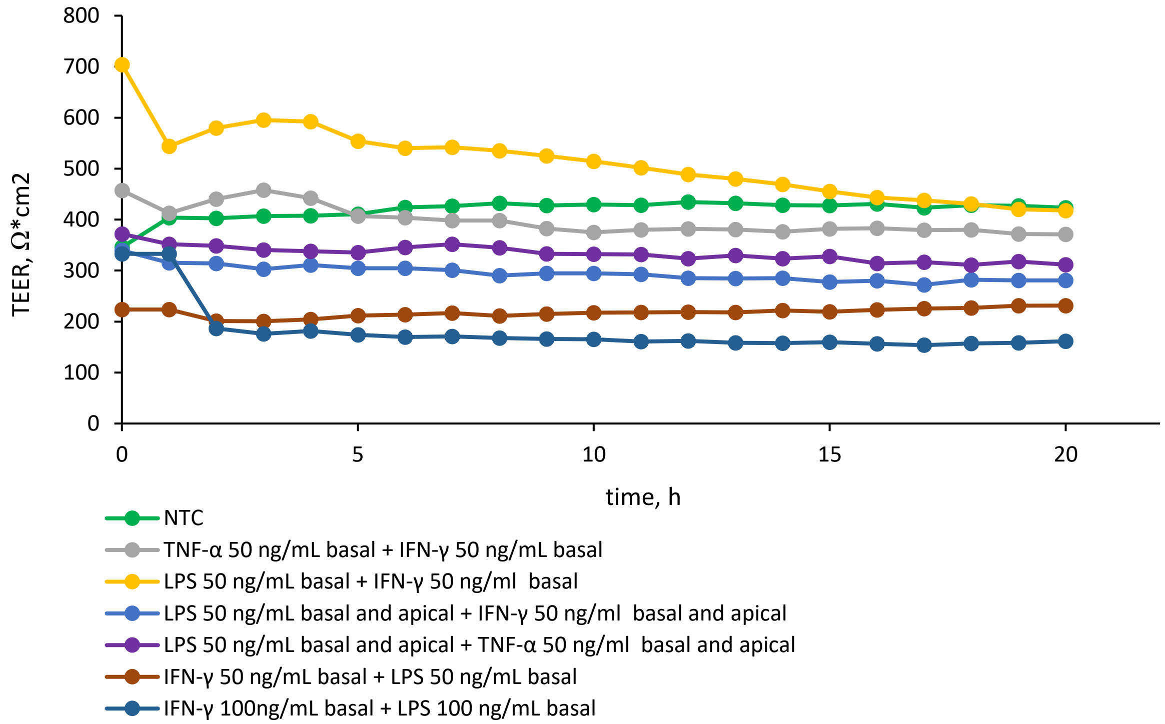

Figure S4. Representative TEER profiles of Caco-2 monolayers after exposure to various tight junction-disrupting factors, added at 0 h. Tested stimuli included combinations of pro-inflammatory cytokines (e.g. TNF-α, IFN-γ) and bacterial components (LPS) at different concentrations and polarities (basal/apical). NTC – non-treated control. For clarity, representative curves from three independent experiments are shown.

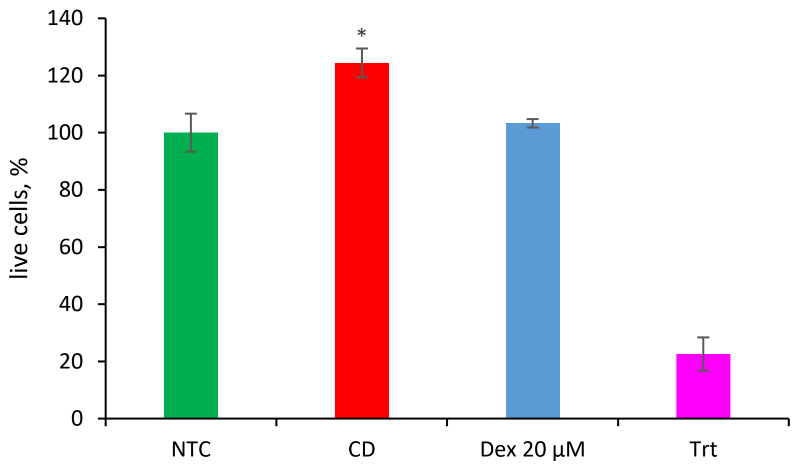

*Figure S5. Effect of a mixture of Tcda/Tcdb A and B (CD, 2.5 and 0.8 ng/mL, respectively) and dexamethasone (Dex,20 µM) on the viability of Caco-2 cells. Triton-X100 (Trt, 0.1% v/v) was used as a positive control. Data were expressed as mean ± SD of three separate experiments conducted in triplicate. Statistical significance *p < 0.05 versus non-treated control (NTC) (Dunnett's post-hoc test).*

Claudin-2

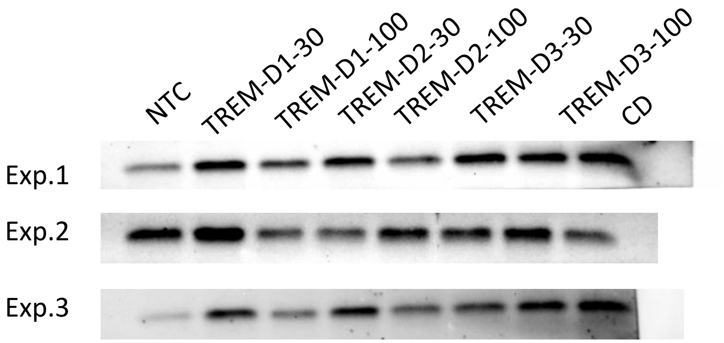
NTC
TREM-D1-30
TREM-D1-100
TREM-D2-30
TREM-D2-100
TREM-D3-30
TREM-D3-100
CD
Exp.1
Exp.2
Exp.3

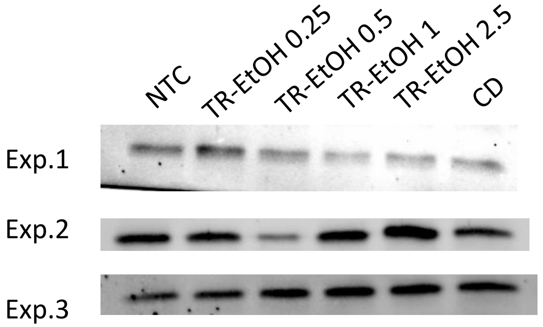
NTC
TR-EtOH 0.25
TR-EtOH 0.5
TR-EtOH 1
TR-EtOH 2.5
CD
Exp.1
Exp.2
Exp.3

Claudin-4

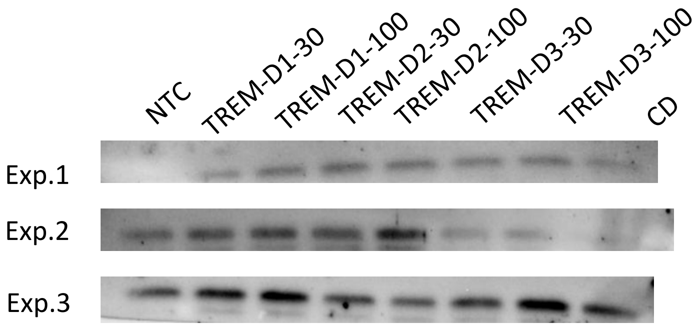
NTC
TREM-D1-30
TREM-D1-100
TREM-D2-30
TREM-D2-100
TREM-D3-30
TREM-D3-100
CD
Exp.1
Exp.2
Exp.3

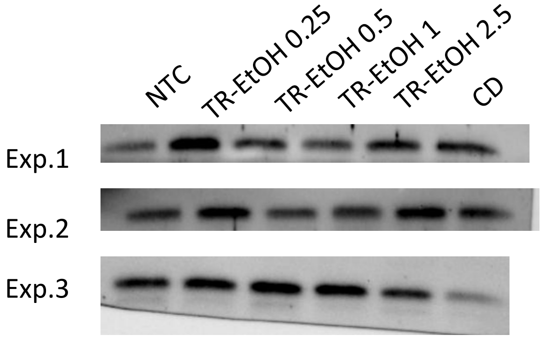
NTC
TR-EtOH 0.25
TR-EtOH 0.5
TR-EtOH 1
TR-EtOH 2.5
CD
Exp.1
Exp.2
Exp.3

Ocludin

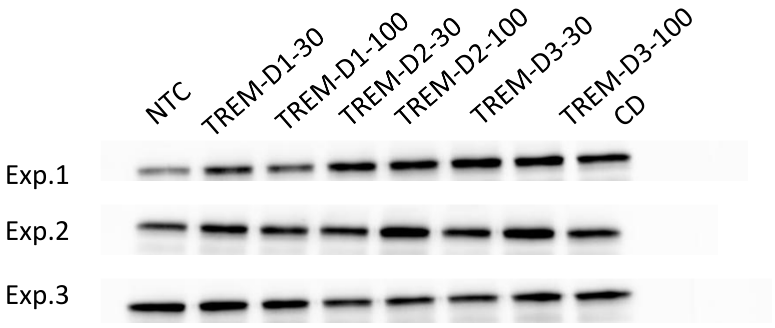
NTC
TREM-D1-30
TREM-D1-100
TREM-D2-30
TREM-D2-100
TREM-D3-30
TREM-D3-100
CD
Exp.1
Exp.2
Exp.3

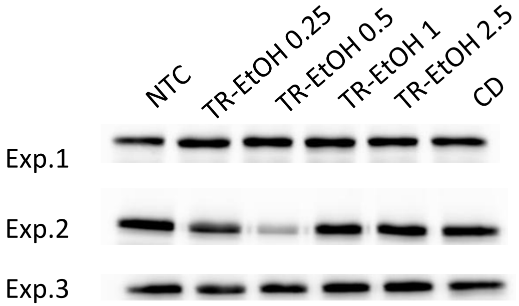
NTC
TR-EtOH 0.25
TR-EtOH 0.5
TR-EtOH 1
TR-EtOH 2.5
CD
Exp.1
Exp.2
Exp.3

β-actin

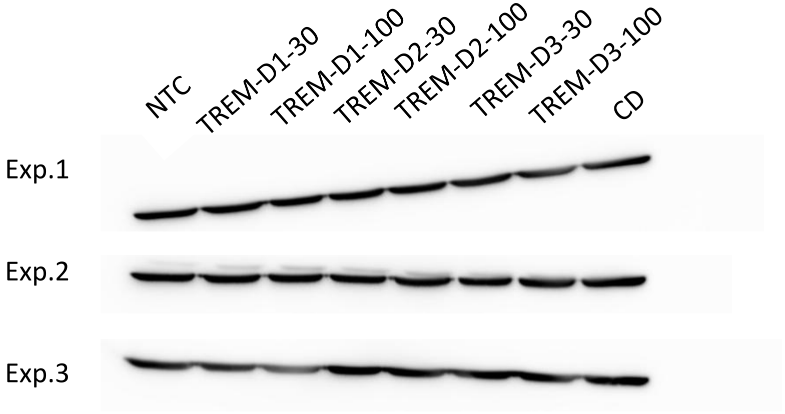

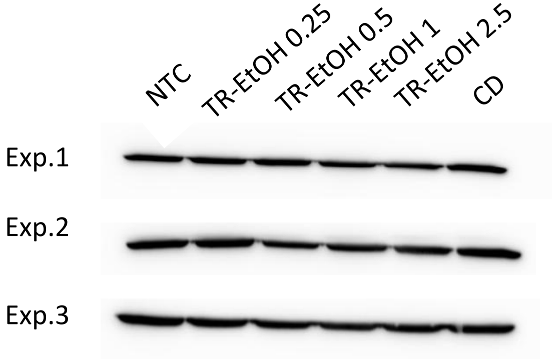

Figure S6. Western blot images showing CLDN-2, CLDN-4, OCLN and β-actin expression in Caco-2 cells pretreated with TR-EtOH (0.25–2.5 mg/mL) and TREMs (equivalent to 1 mg/mL extract) and subsequently exposed to the TcdA/TcdB mixture (2.5 and 0.8 ng/mL, respectively). As a negative control was used MEM with 0.5% DMSO (non-treated control; NTC, and as a positive control was used cells treated with CD.

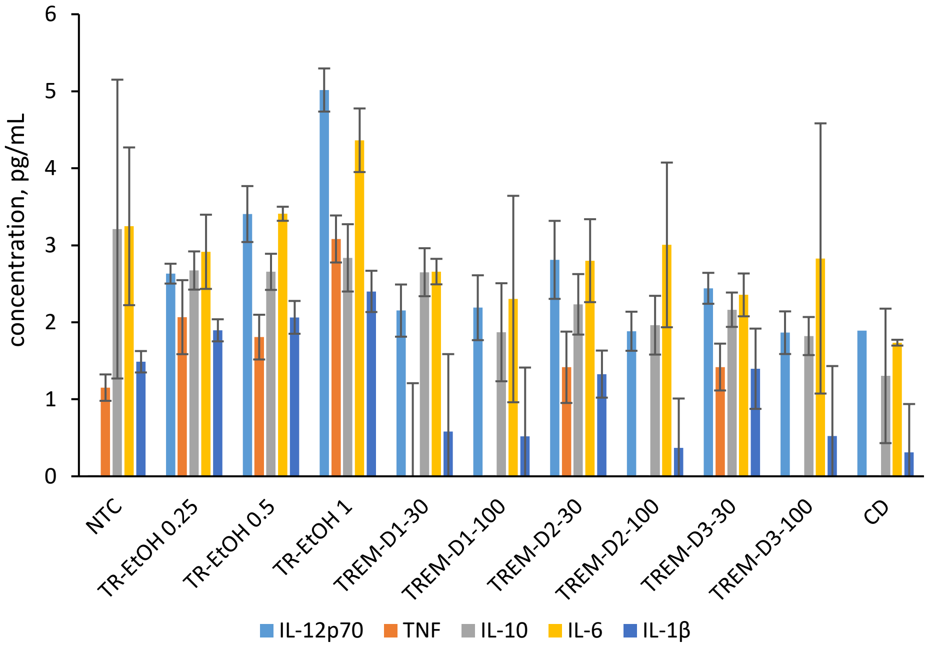

Figure S7. Effect of TR-EtOH (0.25–1 mg/mL) and TREMs (equivalent to 1 mg/mL extract) on IL-12p70, TNF-α, IL-10, IL-6 and IL-1β production in Caco-2 cells stimulated with the TcdA/TcdB mixture (2.5 and 0.8 ng/mL, respectively). As a negative control was used MEM with 0.5% DMSO (non-treated control; NTC, and as a positive control was used cells treated with CD. Data are presented as mean ± SD (n = 3).
